# A Multi-Catalytic Sensing for Hydrogen Peroxide, Glucose, and Organophosphorus Pesticides Based on Carbon Dots

**DOI:** 10.3389/fchem.2021.713104

**Published:** 2021-07-12

**Authors:** Ping Li, Xiang-Ying Sun, Jiang-Shan Shen

**Affiliations:** ^1^ Fujian Provincial Key Laboratory of Featured Biochemical and Chemical Materials, Fujian Province University Key Laboratory of Green Energy and Environment Catalysis, College of Chemistry and Materials, Ningde Normal University, Ningde, China; ^2^ College of Materials Science and Engineering, Huaqiao University, Xiamen, China

**Keywords:** carbon dots (CDs), peroxidase-like catalytic activity, catalytic sensing, H_2_O_2_ and glucose sensing, organophosphorus (OPs) pesticide sensing

## Abstract

In this work, a facile one-pot hydrothermal route was employed to synthesize a series of fluorescent carbon dots (CDs) by using 20 natural amino acids, respectively, as the starting materials. It was found that the CDs synthesized using phenylalanine could possess the intrinsic peroxidase-like activity that could effectively catalyze a traditional peroxidase substrate like 3, 3’, 5, 5’- tetramethylbenzidine (TMB) in the presence of H_2_O_2_ to produce a blue solution; thereby, a catalytic sensing system for H_2_O_2_ has been developed. On the basis of this catalytic reaction, together with the fact that glucose oxidase (GOx) can catalyze the hydrolysis of glucose to generate H_2_O_2_, a sensitive catalytic sensing system for glucose could be further established. Furthermore, based on this catalytic reaction, taken together with the two enzymatic catalytic systems of acetylcholinesterase (AChE) and choline oxidase (CHO), a highly sensitive multi-catalytic sensing system could be successfully developed for organophosphorus (OPs) pesticides such as dimethoate, DDVP, and parathion-methyl. Limit of detections (LODs) of H_2_O_2_ and glucose were estimated to be 6.5 and 0.84 μM, respectively. The limit of detection of the sub-nM level could be obtained for tested dimethoate, DDVP, and parathion-methyl OPs pesticides. The established sensing systems can exhibit good practical application performance in serum and several fruit samples.

## Introduction

Catalysis reaction is an important way to establish extremely sensitive spectral sensing systems. In general, one catalyst can repeatedly participate in certain chemical reactions, resulting in multiple non-fluorescent or low-fluorescence substrate molecules that can be converted into fluorescent species with high quantum yield or multi-colorless substrate molecules that can be converted into colored species with a high molar absorption coefficient in the visible region. In theory, these fluorescent or colored products can function as signal reporters to indicate targets if they can inhibit or facilitate the catalytic reactions *via* certain interactions between targets and catalysts, with high sensitivity. On the basis of these intriguing features, numerous ingenious catalytic sensing systems have been developed to detect a series of important targets including metal ions, biomolecules, and environmental pollutants (Wu and Anslyn, 2004; Bonomi et al., 2011; Vedamalai et al., 2014; Sun et al., 2016; Wu et al., 2017; Wu et al., 2019). For example, Eric V. Anslyn’s group developed a sensitively catalytic sensing system based on a fluorogenic reaction catalyzed by metal ions (Wu and Anslyn, 2004). At first, a polyaza cyclam (PAC) was employed as the deactivating ligand to pre-complex the catalyst Pd (II) to form Pd (II)-PAC. Then, upon the pretreatment of PAC with Cu (II), which has a larger affinity toward PAC than Pd (II), the deactivating ligand is only able to fractionally capture Pd (II), thereby leaving an equal amount of Pd (II) to be reduced to the Pd (0) catalyst in a Heck coupling cycle. Each equivalent of Cu (II) should free up a set amount of catalyst; thereby, the fluorescence is catalytically “turned on” due to creating fluorescent species in the Heck reaction. Leonard J. Prins’ group developed a novel way to probe the enzymatic activity by detecting the binding events between oligoanions and the surface of monolayer-coating Au nanoparticles terminated by triazacyclononane·Zn^2+^ (TACN·Zn^2+^) complexes. (Bonomi et al., 2011) An activated RNA model substrate, 2-hydroxypropyl-4-nitrophenyl phosphate (HPNP), was employed as the substrate catalyzed by the cooperative interaction of two neighboring TACN·Zn^2+^ complexes to generate a reporter molecule, the *p*-nitrophenolate anion. Oligoanions such as ATP acted as competitive inhibitors for the binding between HPNP and the TACNZnII head and could turn off the catalytic activity of the system. Yet, certain enzymes were introduced to cleave oligoanions into smaller anions, which would restore the catalytic activity of Au nanoparticles to indicate the enzymatic activity. On the basis of the reported works above, not only is developing novel catalytic systems with high catalytic activity important but also we believe that developing novel catalytic sensing systems by introducing multi-catalytic reactions will substantially improve the sensitivity.

We know that carbon dots (CDs) are one kind of newly emerging carbon nanomaterial (CNM), with extremely small sizes (∼ less than 5 nm). CDs have demonstrated unique photochemical properties (Yang et al., 2017), good biocompatibility (Zhuo et al., 2015), low toxicity (Li et al., 2021), excellent stability in aqueous solution (Cayuela et al., 2016), and facile synthesis procedures (Zhu et al., 2015).

In general, photoluminescent CDs can be prepared by using N, S, or other heteroatom-containing organic compounds as reactants (Jiang et al., 2014; Chang et al., 2016; Deng et al., 2018; Hou et al., 2018; Meng et al., 2018; Prekodravac et al., 2019; Wu and Tong, 2019; Yao et al., 2019; Zheng et al., 2019; Hallaj et al., 2020; Ji et al., 2021). Natural amino acids containing N, O, or S heteroatoms, as building blocks of proteins, can show some favorable merits, such as being biocompatible, plentiful, and low cost, which makes them ideal precursors for synthesizing CDs. Karfa and coworkers developed heteroatom-doped CDs using various amino acids *via* a one-step hydrothermal synthesis strategy for sensing of Cd^2+^/Fe^3+^, cell imaging, and showing antibacterial activity toward *E. coli* and photocatalytic activity toward H_2_O_2_ (Karfa et al., 2015). He et al. synthesized N-CDs by using a one-pot microwave-assisted hydrothermal method and using histidine as the reactant for *in vivo* imaging and labeling (Huang et al., 2014). Zeng’s group developed N and S co-doped CDs with orange luminescence by using L-serine with L-cysteine as the reactants for the imaging of peritoneal macrophages of mice (Zeng et al., 2015). Pandit et al. used an array-based sensing method to detect several proteins by employing CDs prepared from the pyrolysis of citric acid in the presence of various amino acids under hydrothermal conditions (Pandit et al., 2019). Sahiner synthesized N- and S-doped CDs by employing the microwave technique and using five kinds of amino acids as the reactants (Sahiner et al., 2019). It is interesting that CNMs were recently found to have excellent peroxidase-like activity and were further employed for sensing a series of important biotargets, such as H_2_O_2_, glucose (Lin et al., 2015), nucleic acid, phosphate, DNA, disease biomarkers, and so on (Shen and Xia, 2014; Niu et al., 2015; Weng et al., 2015; Pandit et al., 2019; Sahiner et al., 2019; Chu et al., 2020; Wei et al., 2020).

Therefore, in this work, following the studies above, a facile one-pot hydrothermal method was employed to prepare CDs by using natural amino acids as the reactants. Compared with other amino acid–based CDs, Phe-based CDs (CDs prepared by using Phe as the reactant) exhibited the more outstanding peroxidase-like activity toward TMB in the presence of H_2_O_2_. Therefore, a simple, highly selective, and sensitive colorimetric assay for the detection of H_2_O_2_ and glucose was developed. Furthermore, this was successfully applied for sensing dimethoate, parathion-methyl, and dichlorvos by introducing additional catalytic reactions. The multi-catalytic sensing system established in this work could provide an excellent platform for biological and chemical target analytes due to cascade reaction, and it is expected to expand its range of applications.

## Experimental

### Reagents

Tris (hydroxymethyl) aminomethane (Tris, BR), HCl (36–38%, AR), H_2_O_2_ (30%, AR), NaOAc (99%, AR), HOAc (99.5%, AR), and D-glucose (AR) were purchased from GuoYao (Shanghai, China). TMB (99%, BR), phenylalanine (L-form, 99%, AR), glucose oxidase (AR), parathion-methyl, and dichlorvos (DDVP, 1,000 μg l^−1^ in methanol) were obtained from Aladdin Industrial Corporation (Shanghai, China). Glucose oxidase (GOx, 250 kU mg^−1^, from *Aspergillus niger*) was purchased from Sangon Biotech (Shanghai, China). Choline oxidase (CHO, from *Alcaligenes* sp.), acetylthiocholine chloride (ATCh), acetycholinesterase (AChE, from *Electrophorus electricus*), and dimethoate (5,000 μg ml^−1^ in methanol) were purchased from Sigma-Aldrich. Milli-Q ultrapure water (18.2 MΩcm^−1^, Millipore, United Kingdom) was used to prepare all aqueous solutions.

### Instruments

All PL spectra measurements were performed using a Hitachi F-7000 PL spectrophotometer (Hitachi Co., Ltd.) under an excitation and emission slit of 2.5 and 5.0 nm, respectively. UV–vis absorption spectra experiments were carried out using a Shimadzu UV-2600 PC spectrophotometer. High-resolution transmission electron microscopy (HRTEM) images were collected using a Tecnai F20 microscope (Philips-FEI Co., Holland) operated at 200 KV. The X-ray photoelectron spectra (XPS) were acquired using an ESCALAB 250Xi (Thermo Fisher Scientific, United States), where the analysis chamber was 1.5 × 10^–9^ mbar and the X-ray spot was 500 μm. The time-resolved fluorescence decay curves were obtained using an Edinburgh FLS920 PL spectrometer (Edinburgh, United Kingdom) with a 370-nm nano-LED as the excitation source. The electron spin resonance (ESR) spectra were obtained using a Bruker ESR 300 E with a microwave bridge (receiver gain, 1 × 10^5^; modulation amplitude, 2 Gauss; microwave power, 10 mW; modulation frequency, 100 KHz).

### Synthesis of Carbon Dots

On the basis of a facile one-pot hydrothermal approach, the CDs were prepared by using amino acids as the reactants; for example, phenylalanine was used to synthesize CD-phenylalanine. The detailed procedures of synthesizing CDs are shown in SI†. The as-prepared CD solutions were stored at 4°C before further usage.

### Measurement of PL QY and PL Lifetime of Carbon Dots

The photoluminescence quantum yield (PL QY) of various CDs was determined by using quinine sulfate as a reference (the detailed procedure is shown in SI†).

The PL lifetime of CDs was assessed *via* time-resolved fluorescence measurements. Based on a nonlinear least-squares analysis, and according to the following Eq. 1 (Huang et al., 2014), the decay trace was fitted by using bi-exponential functions Y (t):

$$\text{Y}\left(\text{t}\right)=B_{1}\text{exp}\left(-t/\tau_{1}\right)+B_{2}\text{exp}\left(-t/\tau_{2}\right),$$ (1)

in which B_1_ and B_2_ are the corresponding fractional contributions of the time-resolved decay lifetimes of *τ*_1_ and *τ*_2_, respectively. The average lifetime of the decay process could be calculated by using Eq. 2 (Niu et al., 2015) as follows:

$$\tau_{av}=\frac{{\text{B}}_{1}{\text{τ}}_{1}^{2}+{\text{B}}_{2}{\text{τ}}_{2}^{2}}{{\text{B}}_{1}\tau_{1}+{\text{B}}_{2}\tau_{2}}.$$ (2)

### Measurement of Peroxidase-Like Activity of Carbon Dots

The peroxidase-like activity of the CDs was evaluated by using TMB as the substrate in the presence of H_2_O_2_. 20 μL 6 mg ml^−1^ CDs, 400 μL 5 mM TMB, 300 μL 45 mM H_2_O_2_, and a certain volume of 0.2 moll^−1^ NaOAc–HOAc buffer solution with a pH value of 4.2 were mixed to obtain a solution with a total volume of 3 ml. The mixed solution was monitored by recording the reaction time–dependent UV–vis absorbance change at 652 nm with a time interval of 30 s. To investigate the effects of pH, temperature, and the concentration of H_2_O_2_ and CDs, the peroxidase-like activity of CDs was measured under the following conditions: temperature was varied from 25 to 65°C, pH was adjusted from 3.60 to 8.85, H_2_O_2_ concentration was varied from 22.5 μM to 0.315 mM, and CD concentration was changed from 0 μg ml^−1^ to 80 μg ml^−1^, respectively.

### H_2_O_2_, Glucose, and Pesticide Sensing

#### H_2_O_2_ Sensing

A typical colorimetric analysis for H_2_O_2_ was achieved as follows: first, 400 μL 5 mM TMB, 20 μL 6 mg ml^−1^ CD solution, and various volumes of H_2_O_2_ stock solution were added to a certain volume of 0.2 mol.l^−1^ NaOAc–HOAc buffer solution with a pH value of 4.2 to yield a solution with a total volume of 3 ml, and the resulting final concentration of H_2_O_2_ was 22.5, 45, 67.5, 90, 135, 180, 270, and 315 μM, respectively. Finally, the mixed solution was used for measuring reaction time–dependent UV–vis absorbance at 652-nm curves by using a UV-2600 spectrophotometer. Average values were obtained by measuring three parallel samples.

#### Glucose Sensing

The experimental procedure for sensing glucose was as follows: 1) various volumes of glucose stock solution and 6 μL 4 mg ml^−1^ GOx stock solution were mixed and incubated in a water bath at 37°C for 0.5 h. 2) 400 μL 5 mM TMB, 20 μL 6 mg ml^−1^ CD stock solution, and 1.5 ml 0.2 M NaOAc–HOAc buffer solution with a pH value of 4.2 were added to 200 μL of the reaction solutions above. 3) Then, the mixed solutions were diluted to 3 ml with ultrapure water, and the resulting solutions were incubated at 35°C for 15 min. 4) The UV–vis absorbance of the resulting reaction solution was then measured at 35°C, and the wavelength was set at 652 nm. Average values were obtained by measuring three parallel samples.

#### Pesticide Sensing

The working solutions of dimethoate, DDVP, and parathion-methyl were freshly prepared in isopropanol by diluting corresponding dimethoate, DDVP, and parathion-methyl stock solutions to produce a series of concentrations. Sensing of pesticides was carried out as follows: 1) 10 μL pesticide working solutions of various concentrations, 10 μL 50 unit ml^−1^ AchE, and 80 μL 50 mM Tris-HCl buffer solution with a pH value of 7.4 were mixed, and the resulting solution was incubated at 37°C for 10 min. 2) 10 μL 50 mM ATch and 10 μL 50 unit ml^−1^ CHO were mixed with 80 μL 50 mM Tris-HCl buffer solution with a pH value of 7.4, which were added to the resulting solution of (Eq. 1), and the mixed solution was incubated at 37°C for 15 min. 3) The resulting solution of (2) was then added to 1.5 ml 200 mM NaOAc–HOAc buffer solution with a pH value of 4.2 containing 400 μL 5 mM TMB solution and 20 μL 6 mg ml^−1^ CD solution. The mixed solution was diluted to 3 ml with ultrapure water, and the mixture was incubated for another 15 min at 35°C to allow for color development. 4) The absorbance of the resulting solution of (3) at the 652-nm wavelength was measured. The final concentrations of ATCh, AChE, and CHO in this sensing system were 0.167 mM, 0.167 unit ml^−1^, and 0.167 unit ml^−1^, respectively. Average values were obtained by measuring three parallel samples.

### Kinetics Analysis

The steady-state kinetic measurements were carried out by recording the time-dependent absorbance at the 652-nm wavelength with an interval of 30 s. The initial rates were calculated to be plotted with varying concentrations of TMB and a fixed concentration of H_2_O_2_, and *vice versa*. When the TMB or H_2_O_2_ concentration was changed, the corresponding concentrations of H_2_O_2_ and TMB were fixed at 4.5 and 0.67 mM, respectively. The apparent kinetic parameters were calculated based on the Michaelis–Menten constant (eq. 3) and the Lineweaver–Burk plot (Eq. 4) (Dutta et al., 2012; Biswas et al., 2016) as follows:

$$\text{V}={\text{V}}_{max}\left[S\right]/\left(K_{m}+\left[S\right]\right),$$ (3)

$$1/\text{V}=\left(K_{m}/V_{max}\right)/\left(1/\left[S\right]\right)+\;\left(1/V_{max}\right),$$ (4)

where V is the initial rate, V_max_ is the maximum reaction rate, (s) is the concentration of the substrate (TMB or H_2_O_2_), and K_m_ is the Michaelis constant, which approximates the affinity of the enzyme for the substrate.

### Sample Analysis

The detection of glucose in fruit juice and human serum samples was performed as follows: 1) sample pretreatment: the fruit (apple, orange, and watermelon) juice samples were centrifuged at a rate of 10^4^ rpm for 40 min. The serum sample was treated by ultrafiltration on a 10-KDa ultrafiltration membrane and then centrifuged at a rate of 10^4^ rpm for 40 min. 2) Sample detection: 194 μL of the treated sample solution was added to a solution containing 6 μL 4 mg ml^−1^ GOx. Then, the mixed solution was kept at 37°C for 0.5 h. Subsequently, 400 μL 5 mM TMB, 20 μL 40 μg ml^−1^ Phe-CDs, and 1.5 ml 0.2 moll^−1^ NaOAc–HOAc buffer solution with a pH value of 4.2 were added to 200 μL of the reaction solutions above. The following procedure was carried out in accordance with the procedures of *Glucose Sensing*. In the control experiments, 5 mM maltose, 5 mM fructose, and 5 mM lactose were used to replace glucose, while other experimental conditions remained unchanged. In addition, comparison of the glucose concentrations obtained using the method above and those provided by the hospital was carried out.

The detection of pesticides in fruit peel samples was performed as follows: 1) sample pretreatment: the sample pieces were extracted for 2 min by using 5 ml 50 mM Tris-HCl buffer solution with a pH value of 7.4 per 1 g of sample. The resulting solution was poured out and then placed for ca. 3–5 min. 2) Sample detection: 10 μL extracted solution was further dissolved in 80 μL 50 mM Tris-HCl buffer solution with a pH value of 7.4 containing 10 μL 50 unit ml^−1^ AChE, and the mixed solution was incubated at 37°C for 10 min. The subsequent procedure was in accordance with (Eq. 2), (Eq. 3), and (Eq. 4) of *Pesticide Sensing*. Average values were obtained by measuring three parallel samples.

### Recovery Experiments of Glucose and Pesticides in Spiked Practical Sample

Apple, orange, and watermelon juice was chosen as a practical sample for glucose recovery, respectively. Apple, orange, and watermelon peel was chosen as a practical sample for pesticide recovery, respectively. The sample pretreatment was finished for the practical samples above, according to section 2.8.

## Results and Discussion

### Characterization of Carbon Dots

**FIGURE 1:**
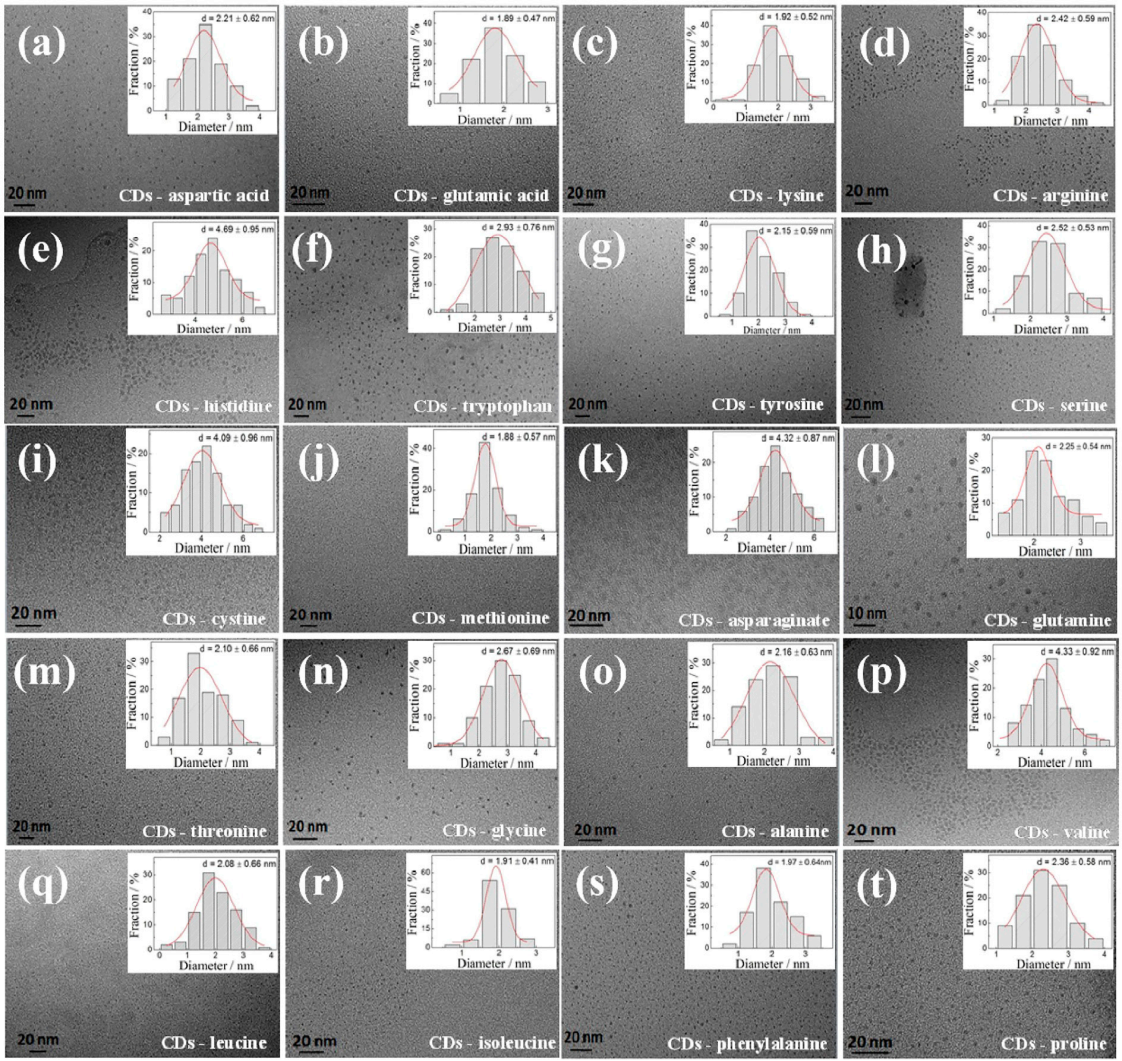
HRTEM images of different CDs. Inset: the corresponding particle size distribution histograms of CDs from 100 nanoparticles.The CDs a-t: CDs-aspartic acid, CDs-glutamci, CDs-lysine, CDs-arginine, CDs-histidine, CDs-tryptophan, CDs-tyrosine, CDs-serine, CDs-cystine, CDs-methionine, CDs-asparaginate, CDs-glutamine, CDs-threonine, CDs-glycine, CDs-alanine, CDs-valine, CDs-leucine, CDs-isoleucine, CDs-phenylalanine and CDs-proline, respectively.

Figure 1 shows a TEM image of the as-prepared CDs, and Figure 1 (inset) shows the particle diameter distribution of the CDs obtained by measuring the particle sizes of 100 nanoparticles. These as-prepared CDs were well dispersed with an average diameter of less than 5 nm. Among all tested CDs, the biggest size was found in the CD-histidine sample with a diameter of ca. 4.69 ± 0.95 nm and the smallest size occurred in the CD-methionine sample with a diameter of *ca*. 1.88 ± 0.57 nm. For CD-phenylalanine, a small diameter of ca. 1.97 ± 0.64 nm was also obtained. The sizes of CDs prepared from other amino acids varied in the range of *ca.* 1–5 nm. However, it is hard to rationally establish a relationship between the size of as-prepared CDs and the kinds of amino acids used at this stage because of the hydrophobic interaction, π–π stacking interaction of phenyl rings, and the effect of heteroatoms such as N and S atoms of the amino acids used on the size, which is a quite complex process. The photographs of CD solutions are shown in Supplementary Figure S1.

UV–vis absorption and PL spectra of the as-prepared CDs are recorded in Supplementary Figure S2 and Supplementary Figure S3. These UV–vis absorption spectra display significant absorption peaks centered at ca. 250–280 nm, quietly different from those of unreacted amino acids. The PL QYs of as-prepared CDs are recorded in Supplementary Table S1. The PL QY of CD-arginine, CD-threonine, CD-aspartic acid, and CD-asparagine was calculated to be 0.15, 0.15, 0.14, and 0.13, respectively, higher than that of other tested CDs in this work. The photoluminescence lifetime of different CD_S_ is shown in Supplementary Figure S4. The average lifetime of CD-threonine was 14.23 ns, which is longer than that of other tested CDs. The average PL lifetime of CDs is also summarized in Supplementary Figure S1.

### Peroxidase-Like Catalytic Activity of Carbon Dots

**FIGURE 2:**
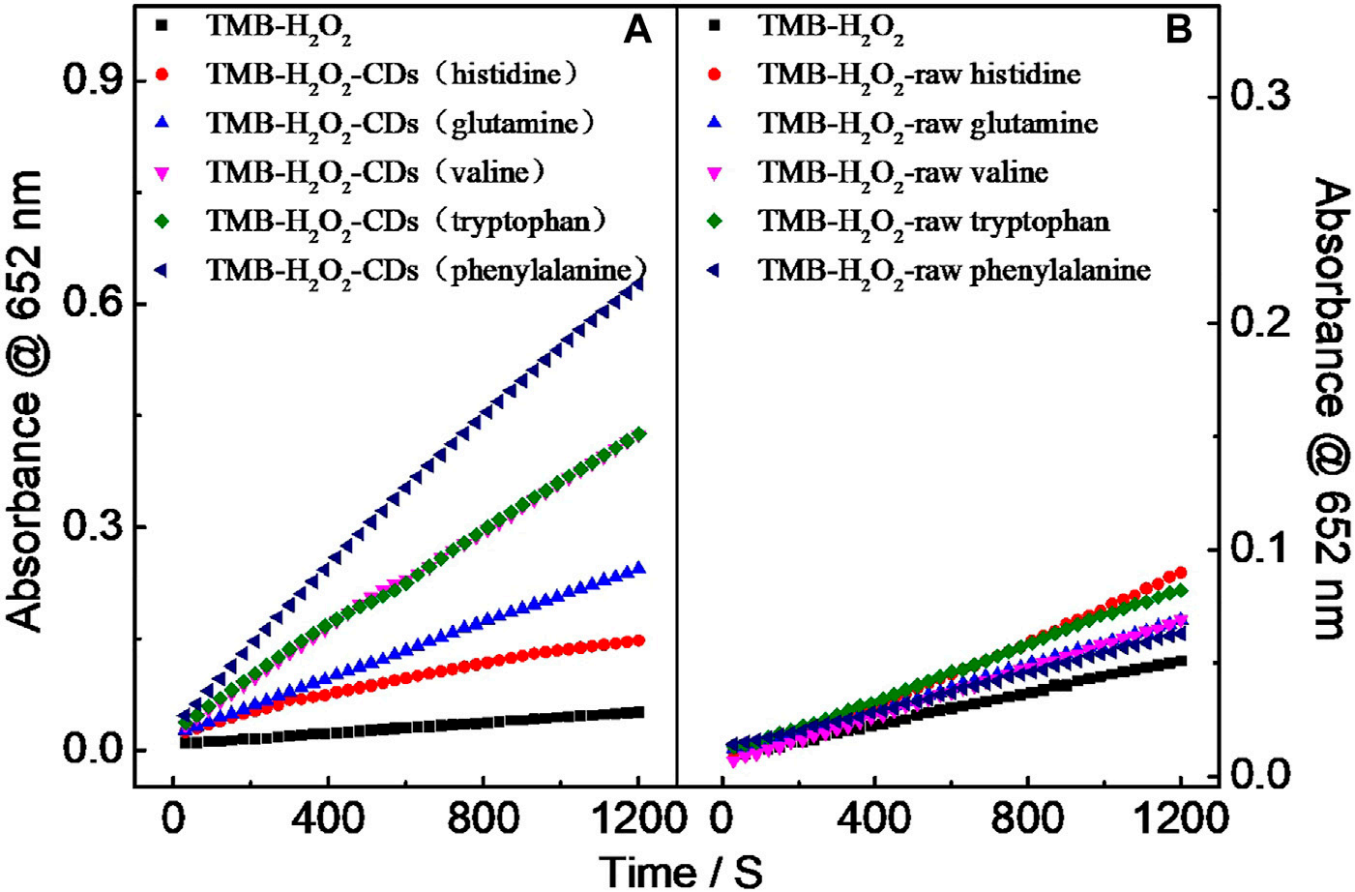
Relationship curves of Ox TMB of absorbance at the 652-nm wavelength **(A)** and time in reaction systems **(B)**.

When TMB and the as-prepared CDs were mixed in the presence of H_2_O_2_, it was surprisingly found that the color of the mixed solution gradually turned to blue from the original colorless while extending the reaction time. Reaction time–dependent UV–vis absorption spectra further showed that CDs prepared from histidine, glutamine, valine, phenylalanine, and tryptophan could exhibit favorable catalytic activity toward TMB in the presence of H_2_O_2_ (Figure 2A). Compared with the as-prepared CDs, no color change of unreacted amino acids toward TMB in the presence of H_2_O_2_ could be observed (Figure 2B). Among these tested CDs, CD-phenylalanine demonstrated the highest catalytic activity. In order to explore the excellent peroxidase-like catalytic behavior of CD-phenylalanine, we selected the aforementioned three kinds, CD-tryptophan, CD-valine, and CD-phenylalanine, with strong catalytic performance as the research object, and discussed the factors that may affect the peroxidase-like catalysis. Table 1 shows the catalytic influence factors (size, functional groups, and C content) of CDs. It can be seen from Table 1 that the size of the CD-phenylalanine is the smallest, and the large specific surface area provides a good active site for catalytic behavior. Phenylalanine contains a benzene ring, which may accumulate π–π with the biphenyl on the TMB molecule and close the distance with the TMB molecule. In addition, C content of CD-phenylalanine is higher, indicating that the internal carbon nucleus formation is better. The literature shows that the catalytic activity of nanomaterials comes from the action of surface sites and/or internal nuclei, and the catalytic role of internal nuclei is more important (Chen et al., 2015). CD-phenylalanine was chosen for further detailed study.

**TABLE 1:** Catalytic influence factors from CDs with better catalytic performance.

CD samples	Particle size	Functional group	C content (%)
CD-tryptophan	2.93 ± 0.76	Indole ring, −NH_2_ group, and −COOH group	71.53
CD-valine	4.33 ± 0.92	−NH_2_ group and −COOH group	62.83
CD-phenylalanine	1.97 ± 0.64	Benzene ring, −NH_2_ group, and −COOH group	71.65

**FIGURE 3:**
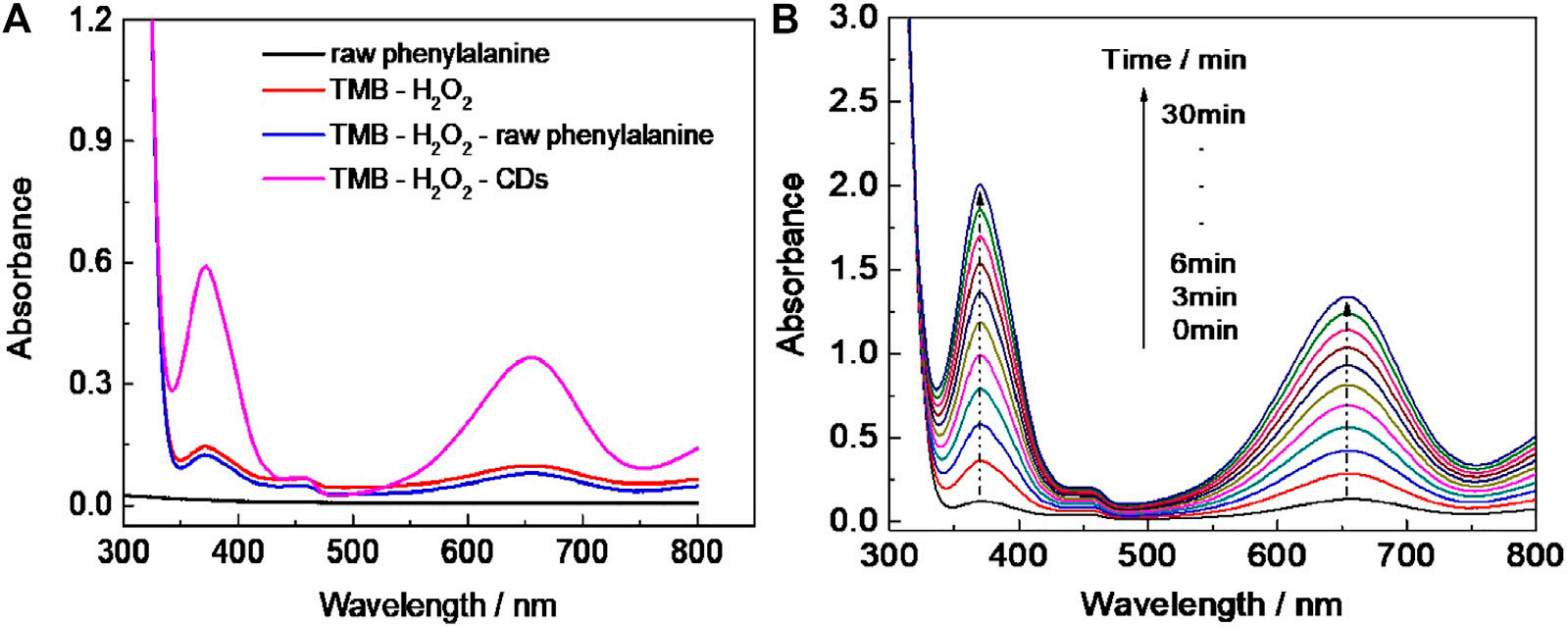
**(A)** UV–vis absorption spectra of the different reaction systems (reaction time = 5 min). **(B)** Time-dependent UV–vis absorption spectra of the TMB–H_2_O_2_–CD-phenylalanine system.

UV–vis absorption spectral study revealed that the new absorption peak of CD-phenylalanine and the TMB mixing solution in the presence of H_2_O_2_ occurred at 652 nm (Figure 3A), and the absorbance gradually increased with extending the reaction time (Figure 3B). In the control experiments, no significant absorbance occurred at 652 nm or increased with increasing the reaction time in the unreacted phenylalanine and TMB mixing solution or only the TMB solution in the presence of H_2_O_2_ (Figure 3A). These observations indicated that CD-phenylalanine possesses peroxidase-like catalytic activity which could catalyze the oxidation reaction of the TMB–H_2_O_2_ system when the experimental conditions were set as follows: CD-phenylalanine = 40 μg ml^−1^, TMB = 0.67 mM, H_2_O_2_ = 4.5 mM, 0.2 M NaOAc–HOAc buffer solution with a pH value of 4.2, and a temperature of 35°C.

### The Effects of Various Experimental Conditions on the Peroxidase-Like Activity

The catalytic activity of CD-phenylalanine was further investigated by varying pH, temperature, and H_2_O_2_ and CD-phenylalanine concentrations.

**FIGURE 4:**
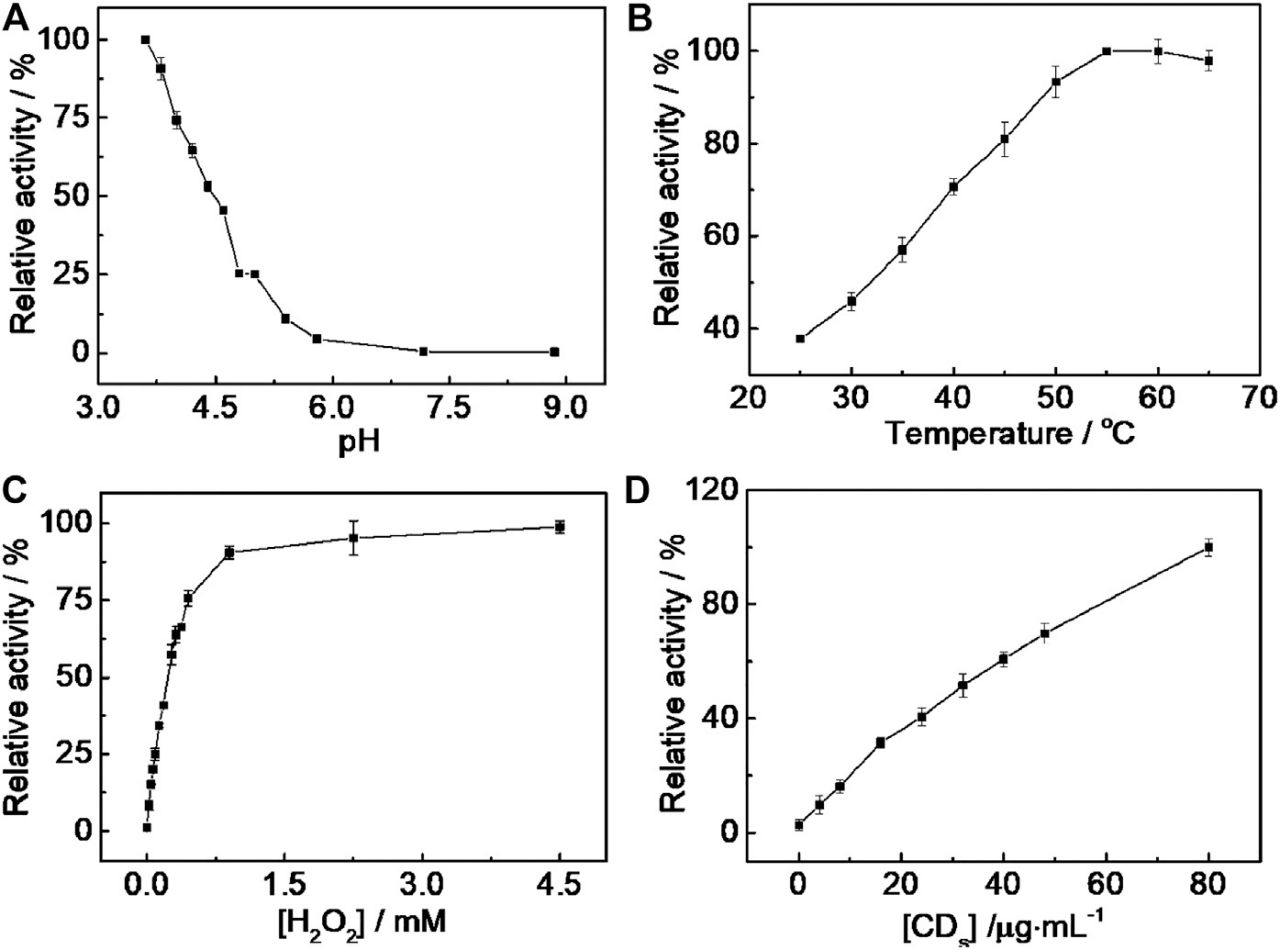
pH **(A)**, temperature **(B)**, H_2_O_2_ concentration **(C)**, and CD-phenylalanine concentration **(D)** dependent peroxidase-like catalytic activity of CD-phenylalanine toward the TMB–H_2_O_2_ system. The maximum point in each curve **(A–D)** was set to 100%.

As shown in Figure 4A, the catalytic activity of CD-phenylalanine decreased with the increasing pH and leveled off when the pH exceeded 5.8, indicating that the catalytic oxidation reaction of TMB occurred easily under acidic conditions. Therefore, 0.2 M NaOAc–HOAc buffer solution with a pH value of 4.2 was taken as the reaction medium for further investigation. The catalytic activity of CD-phenylalanine increased with increasing reaction temperature in the range of 20–65°C, while the catalytic activity slightly decreased when the temperature was higher than 5 °C (Figure 4B), likely due to the decomposition of H_2_O_2_ itself under high temperature. It was obvious that relatively high temperature was unfavorable for this enzymatic-like reaction. The catalytic activity was sharply increased with increasing H_2_O_2_ concentration and reached a saturation state when the H_2_O_2_ concentration reached 0.8 mM (Figure 4C). The effect of the Phe-CD concentration was also investigated, and the result is shown in Figure 4D. The absorbance was gradually increased with increasing Phe-CD concentration. Therefore, 0.2 M NaOAc–HOAc buffer solution with a pH value of 4.2, a temperature of 35°C, 0.67 mM TMB and 4.5 mM H_2_O_2_, and a CD-phenylalanine concentration of 40 μg ml^−1^ were chosen for further experiments.

### Catalysis Mechanism

**FIGURE 5:**
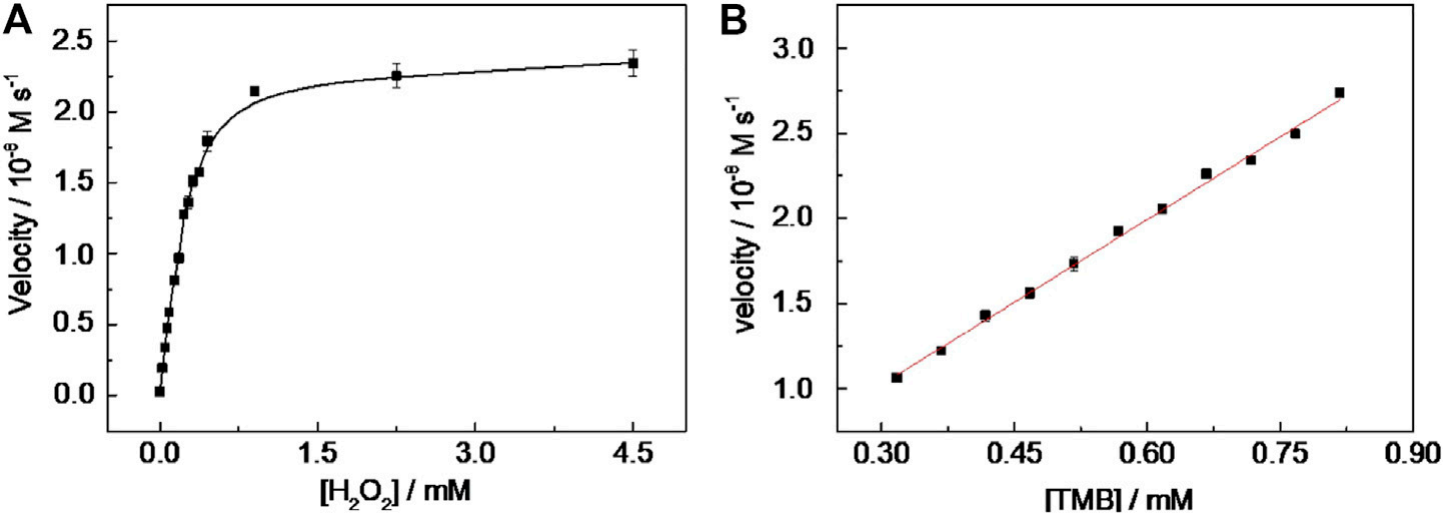
Steady-state kinetic assay of CD-phenylalanine. **(A)** TMB concentration was fixed as 0.67 mM and H_2_O_2_ concentration was varied and **(B)** H_2_O_2_ concentration was fixed as 4.5 mM and TMB concentration was varied.

**FIGURE 6:**
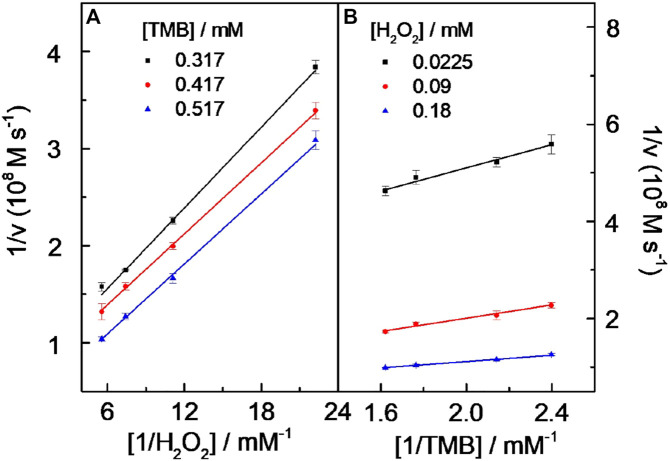
Double reciprocal curves of peroxidase-like catalytic activity of the CDs toward the TMB–H_2_O_2_ system at a fixed concentration of one substrate vs. varying concentrations of the second substrate for TMB **(A)** and H_2_O_2_ **(B)**. Experiment conditions are as follows: 0.2 M NaOAc–HOAc buffer solution with a pH value of 4.2 containing 40 μg ml^−1^ CD solution and a temperature of 35°C. The error bars represent the standard deviation of three parallel measurements.

Under the chosen experimental conditions (viz. CD-phenylalanine = 40 μg ml^−1^, 0.2 M NaOAc–HOAc buffer solution with a pH value of 4.2, 35°C) mentioned by the part above, the reaction rates were calculated *via* a series of kinetics experiments when varying the concentration of one of the substrates and fixing another. Within the suitable concentration range of H_2_O_2_ (Figure 5A) and TMB (Figure 5B), the typical Michaelis–Menten curves could be well fitted. The K_m_ and V_max_ calculated from Lineweaver–Burk plots (Figure 6) are summarized in Table 2. It should be noted that the apparent Michaelis–Menten constant K_m_ valve for CD-phenylalanines using the H_2_O_2_ substrate is less than that using the TMB substrate. This indicated that CD-phenylalanine has a much higher affinity toward H_2_O_2_ than toward TMB.

**TABLE 2:** Apparent kinetic parameters of CD–TMB–H_2_O_2_.

[E](M)	Substrate	Km (mM)	V_max_ (Ms^−1^)	K_cat_ (s^−1^)
3 × 10^−6^	TMB	3.17	1.27 × 10^−7^	4.23 × 10^−2^
3 × 10^−6^	H_2_O_2_	0.25	2.34 × 10^−8^	7.80 × 10^−3^

**FIGURE 7:**
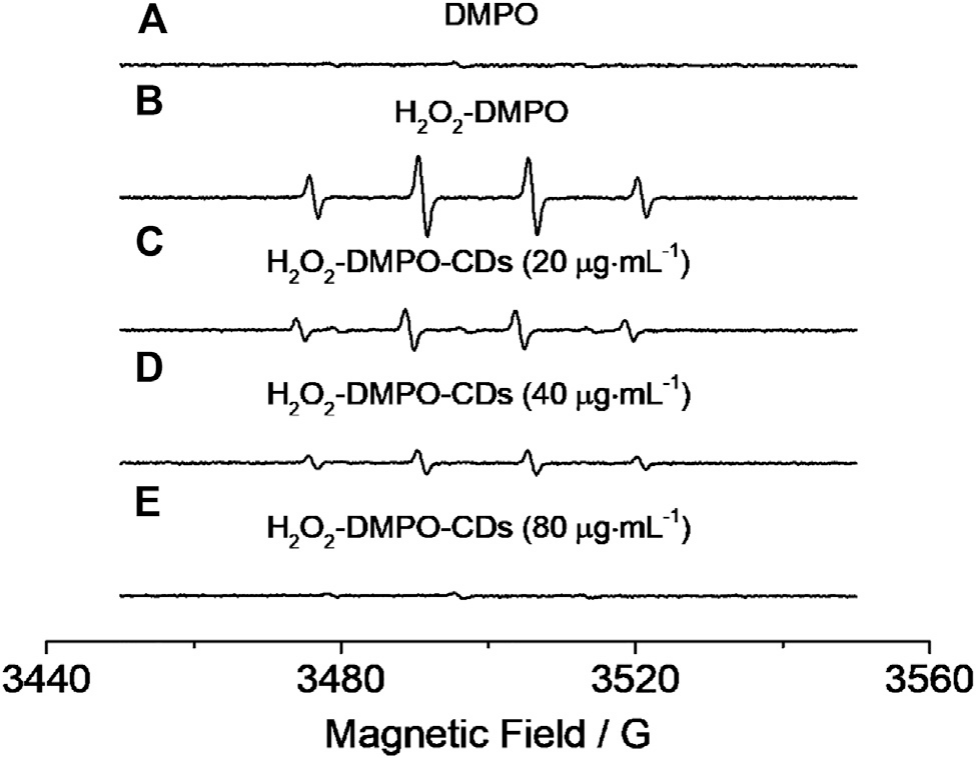
EPR results of **(A)** DMPO, **(B)** H_2_O_2_–DMPO, and **(C–E)** H_2_O_2_–DMPO–CD-phenylalanine under the irradiation of 355-nm UV light.

The parallel lines obtained from Lineweaver–Burk plots (Figure 6) showed that a ping-pong mechanism should be responsible for the reaction of the TMB–H_2_O_2_ system catalyzed by CD-phenylalanine. Therefore, a conclusion could be made that CD-phenylalanine binds and reacts with the first substrate and the first product is released before reacting with the second substrate. The catalysis mechanism of CD-phenylalanine peroxidase-like catalytic activity possibly originates from the decomposition of H_2_O_2_, and then the active species hydroxyl radical (HO·) was generated in the TMB–H_2_O_2_–CD-phenylalanine system. This deduction was supported by the experiments of ESR by adding DMPO, a specific target molecule of HO·, into the TMB–H_2_O_2_–CD-phenylalanine system. ESR experiments were conducted under the following experimental conditions: 4 mM DMPO, 4.5 mM H_2_O_2_ and CD-phenylalanine of various concentrations (20 μg ml^−1^, 40 μg ml^−1^, and 80 μg ml^−1^), and 0.2 M NaOAc–HOAc buffer solution with a pH value of 4.2. The ESR signals were recorded after 8 min of UV light at 355 nm. Figure 7A shows the signal of DMPO in the absence of UV light. The mixed CDs, H_2_O_2_, and DMPO solutions were irradiated under 355-nm UV light. As shown in Figures 7B–E, the signal intensity of the H_2_O_2_/DMPO/CD-phenylalanine system decreased with increasing CD-phenylalanine concentrations. The quad signal peak intensity of DMPO-OH was 1:2:2:1 in the H_2_O_2_/DMPO/CD-phenylalanine system. These results indicated that the catalytic activity of CD-phenylalanine should originate from the generated HO radical, which could catalyze the oxidation reaction of TMB (Dong et al., 2015).

### Sensing of H_2_O_2_ and Glucose

**FIGURE 8:**
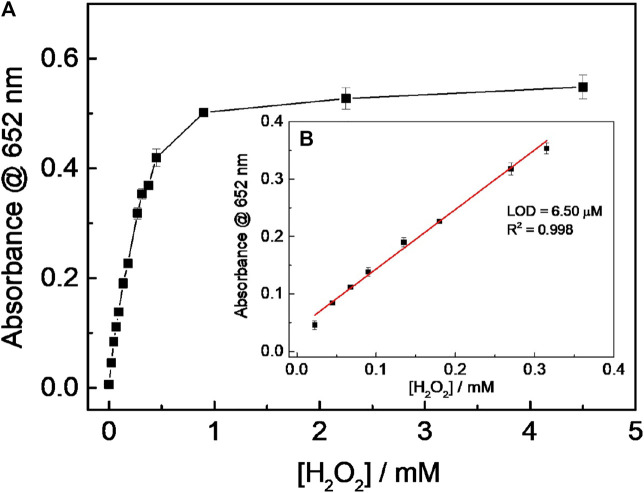
**(A)** Relationship between absorbance at 652 nm and (H_2_O_2_); **(B)** linear fitting curve for (H_2_O_2_) and absorbance at 652 nm. (Inset: pictures were taken 15 min after adding CD-phenylalanine to the solution containing TMB and H_2_O_2_).

Based on the experimental results above, the H_2_O_2_ mixed with various concentrations of 0.67 mM TMB and 40 μg ml^−1^ CD-phenylalanine solution containing 0.2 M NaOAc–HOAc buffer with a pH value of 4.2 was incubated at 35°C for 15 min to record the absorbance at 652 nm. The absorbance was found to be increased with increasing H_2_O_2_ concentration and then reached a saturation profile when the H_2_O_2_ concentration was larger than 0.315 mM (Figure 8A). A good linear relationship between the H_2_O_2_ concentration and the absorbance at 652 nm could be obtained in the H_2_O_2_ concentration range of 0.0225–0.315 mM (Figure 8B), with a limit of detection (LOD) of 6.50 μM. Furthermore, the color variation of the solution was easily observed by the naked eye (Figure 8B inset).

**FIGURE 9:**
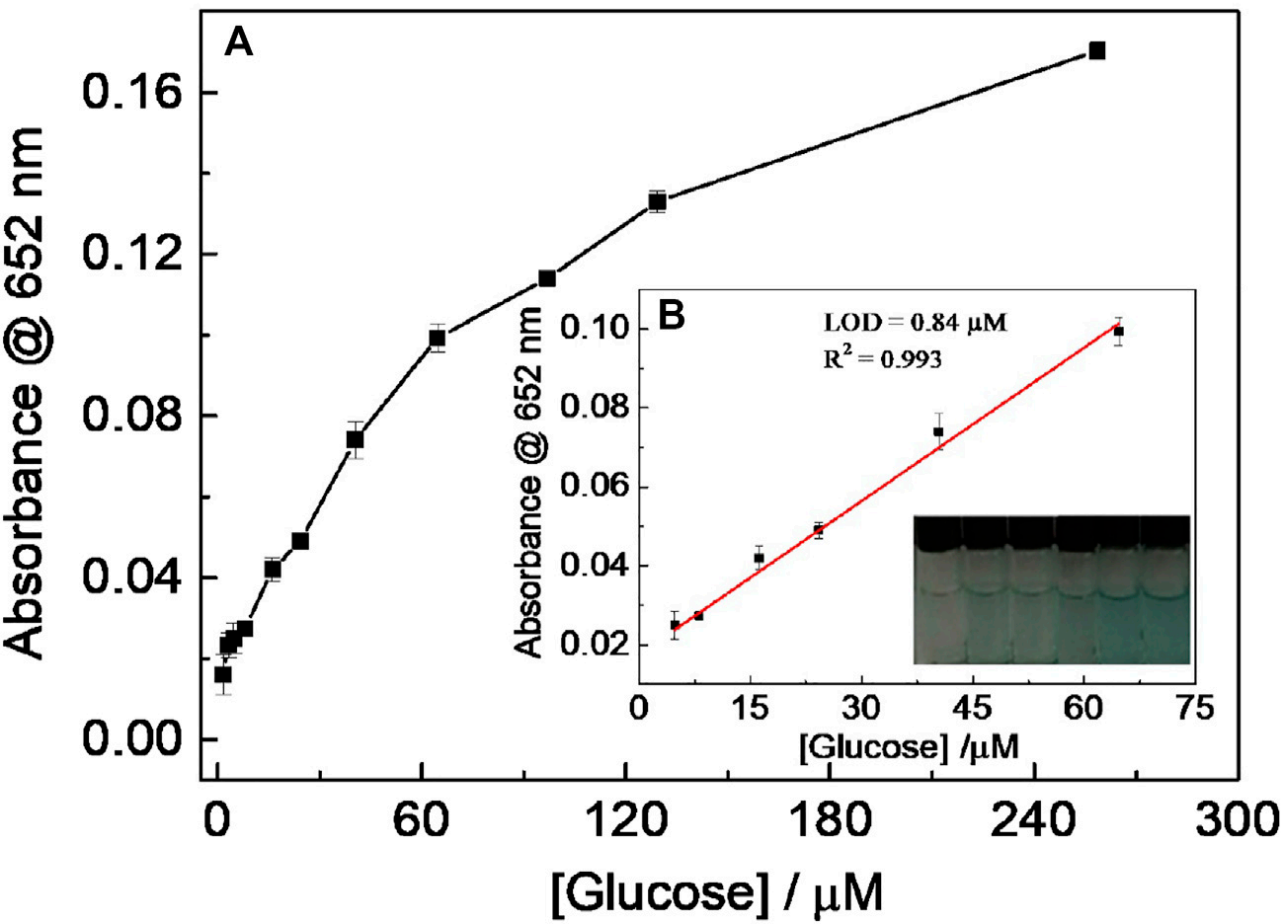
**(A)** Relationship between absorbance at 652 nm and glucose concentration; **(B)** linear fitting curve for glucose detection using the CDs’ peroxidase-like catalytic reaction. (Inset: picture was taken 15 min after adding CD-phenylalanines).

Since H_2_O_2_ is the dominant product of the oxidation reaction of glucose catalyzed by GOx, CD-phenylalanine was also used to probe glucose. When the experimental conditions were set to 0.67 mM TMB, 8 μg ml^−1^ GOx, a reaction temperature of 35°C, 0.2 M NaOAc–HOAc buffer solution with a pH value of 4.2 containing 40 μg ml^−1^ CD-phenylalanine, a method for detecting glucose was established (Figure 9A) Figure 9B shows a typical absorbance at the 652-nm response curve toward glucose concentration in which a good linear fitting could be obtained in the glucose concentration range of 4.85–64.7 μM, with an LOD of 0.84 μM, lower than that in most of the previously reported works in which other nanomaterials were used (Table 3) (Tian et al., 2013; Zhang et al., 2013; Lin et al., 2014; Lin et al., 2015).

**TABLE 3:** Comparison of various methods for detection of the glucose.

Catalyst	Substrate	Method	LOD (*μ*M)	References
g-C_3_N_4_ nanosheets	TMB	Electrochemical	11	Tian et al. (2013)
TiO_2_ nanotubes	TMB	Electrochemical	5	Zhang et al. (2013)
g-C_3_N_4_ nanosheets	TMB	Colorimetric	1	Lin et al. (2014)
GQDs	TMB	Colorimetric	16	Lin et al. (2015)
CDs	TMB	Colorimetric	0.84	This work

The selectivity of sensing glucose was further investigated. The interference experiments were carried out by using 0.2 M NaOAc–HOAc buffer solution with a pH value of 4.2 containing glucose, maltose, fructose, and lactose of certain concentrations, respectively, in which glucose concentration was 1 mM and interfering substances’ concentrations were 5 mM. The experimental results revealed that no detectable signals were obtained for glucose analogs, including maltose, fructose, and lactose (Supplementary Figure S5). Compared with the colorless appearance of the control reaction solution containing glucose analogs, an obvious color change of the reaction solution containing 1 mM glucose, from the original colorless to blue, could be observed (Supplementary Figure S5 inset). Therefore, this method proposed here could show high selectivity toward sensing glucose.

### Sensing Dimethoate, DDVP, and Parathion-Methyl

**FIGURE 10:**
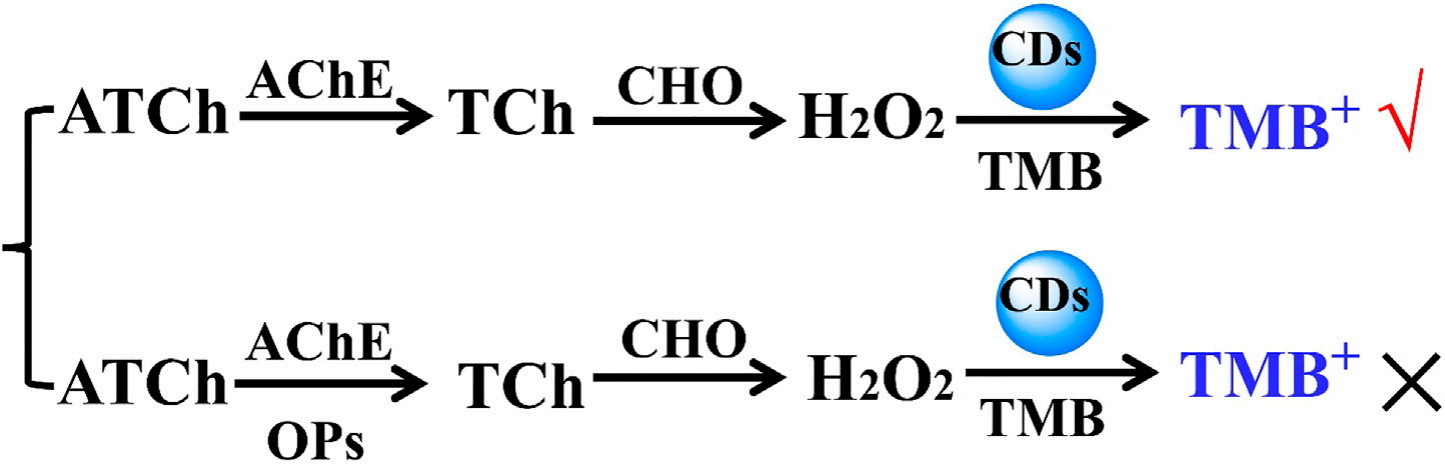
Schematic illustration of detection of OPs based on the photochemical sensing system of the suppressive AChE activity.

Figure 10 shows the sensing scheme for organophosphorus pesticide detection. The hydrolysis reaction of acetylthiocholine (ATCh) can be catalyzed by acetylcholinesterase (AChE) to form thiocholine (TCh), and the oxidation reaction of the resulting TCh can be catalyzed by choline oxidase (CHO) to dominantly generate H_2_O_2_. Therefore, the resulting H_2_O_2_ was expected to be used to achieve the oxidation reaction of TMB catalyzed by CD-phenylalanine. It is well known that organophosphorus and organochlorine pesticides, such as dimethoate, DDVP, and parathion-methyl, can combine with AChE to suppress the catalyzed hydrolysis reaction of ATCh, that is, producing H_2_O_2_ can be prevented in the continuous double enzyme catalyzed reactions of AChE and CHO. This strategy can lead to sensing organophosphorus or organochlorine pesticides, which would be achieved by employing multi-catalytic reactions.

**FIGURE 11:**
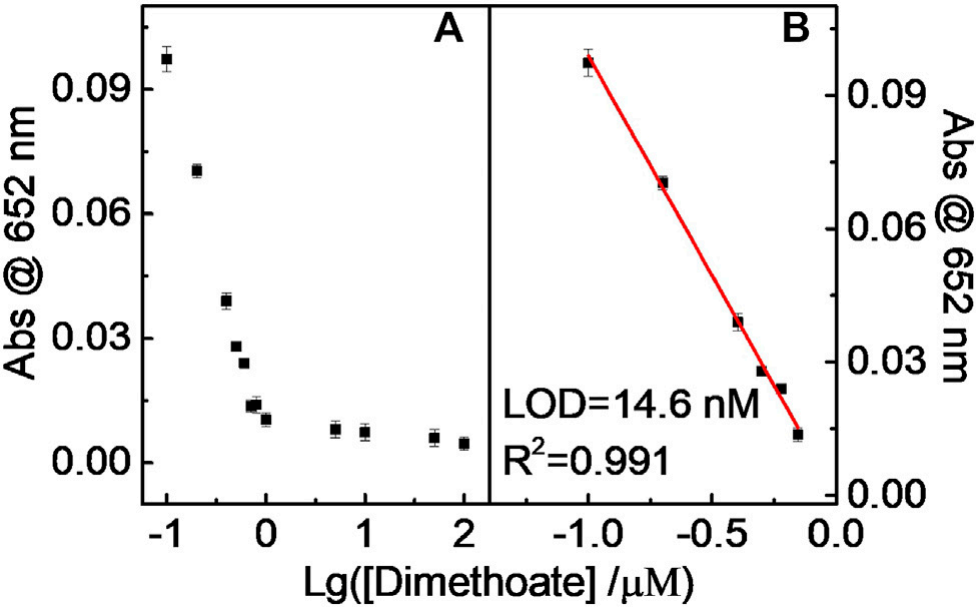
Relationship **(A)** and the linear fitting **(B)** between Abs at 652 nm and Lg (dimethoate).

The dimethoate concentration-dependent UV–vis absorption response curve toward lg (Dimethoate) shows that the absorbance value gradually decreased with increasing dimethoate concentration (Figure 11). Clearly, the oxidation reaction of TMB could be suppressed owing to the presence of a certain amount of dimethoate. Indeed, AChE can catalyze the hydrolysis reaction of ATCh to generate TCh (the first catalytic reaction), capable of acting as the substrate for CHO (the second catalytic reaction). H_2_O_2_ can be produced by the second catalytic reaction. The strong binding of dimethoate and AChE suppressed the catalytic activity of AChE toward the hydrolysis reaction of ATCh, further resulting in less H_2_O_2_. Thus, the amount of introduced dimethoate could be indirectly quantitated.

**FIGURE 12:**
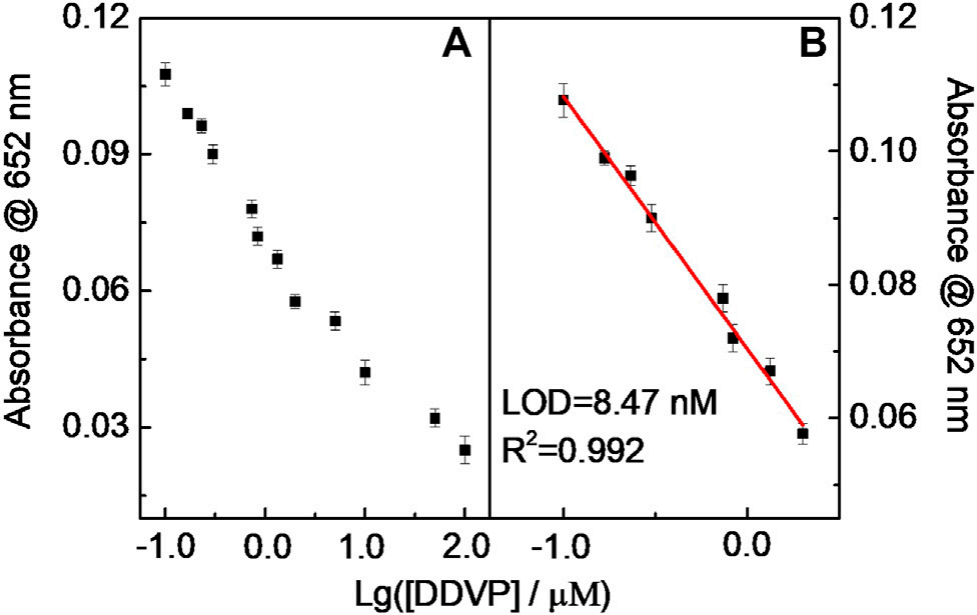
Relationship curve **(A)** and the linear fitting **(B)** between Abs at 652 nm and Lg (DDVP).

**FIGURE 13:**
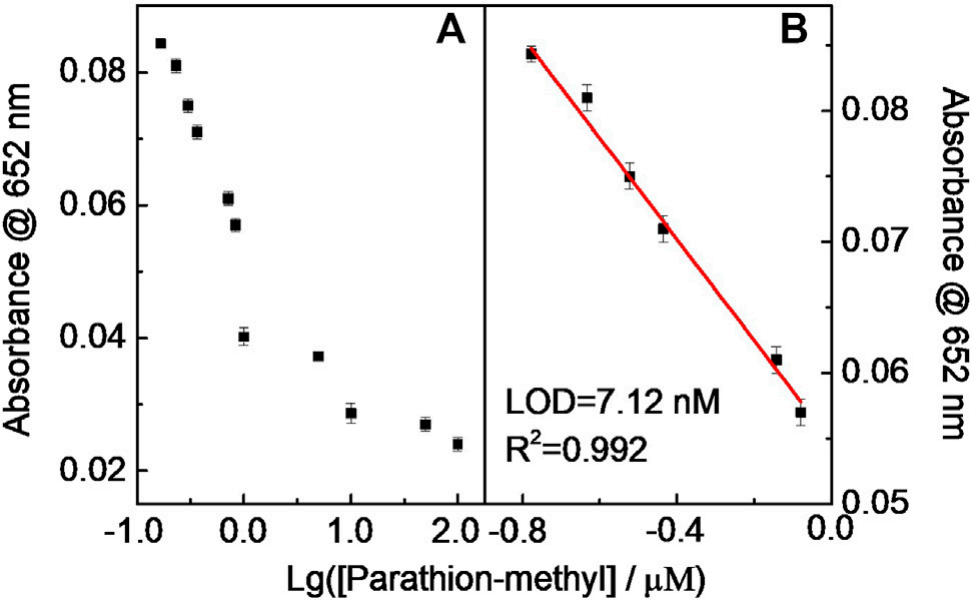
Relationship curve **(A)** and the linear fitting **(B)** between Abs at 652 nm and Lg (parathion-methyl).

Similar results occurred in the DDVP and parathion-methyl cases, that is, the catalytic chromogenic reaction of the TMB–H_2_O_2_–CD-phenylalanine system was suppressed because continuous double enzymatically catalytic systems were introduced (Figure 12 and Figure 13). The sub-nM level of the LOD could be achieved for these tested pesticides. The LODs of parathion-methyl, DDVP, and dimethoate were calculated to be 7.12, 8.47, and 14.6 nM, respectively, lower than that of other reported methods (Table 4) (Pimsen et al., 2014; Zhu et al., 2014; Azab et al., 2015; Yan et al., 2015; Hou et al., 2016; Hsu et al., 2017). The experimental conditions above (Figure 11, Figure 12, and Figure 13) were 40 μg ml^−1^ CD-phenylalanine, 0.67 mM TMB, 0.83 unit ml^−1^ AChE, 0.83 unit ml^−1^ CHO, 0.83 unit ml^−1^ ATCh, 0.2 M NaOAc–HOAc buffer solution with a pH value of 4.2 (TMB–CD catalytic reaction) and 50 mM Tris-HCl buffer solution with a pH value of 7.4 (ATCh-AChE/CHO catalytic reactions), and a temperature of 35°C.

**TABLE 4:** Comparison of our proposed method and the reported cases for pesticide analysis.

Serial number	Pesticide	Method	LOD (nM)	References
1	DDVP	Luminescence	320	Azab et al. (2015)
2	DDVP	Colorimetric	6.70	Pimsen et al. (2014)
3	Dimethoate	Luminescence	13.1	Hsu et al. (2017)
4	Dimethoate	Luminescence	21.8	Hou et al. (2016)
5	Parathion-methyl	Luminescence	48.0	Zhu et al. (2014)
6	Parathion-methyl	Colorimetric	18.0	Yan et al. (2015)
7	DDVP	Colorimetric	8.47	This method
	Dimethoate	Colorimetric	14.6	
	Parathion-methyl	Colorimetric	7.12	

### Practical Application

In order to validate the accuracy of this established sensing system for detecting glucose in practical samples including apple juice, orange juice, and watermelon juice, recovery experiments were then conducted using this proposed method. The experimental results are shown in Supplementary Table S2. The recovery of the method was 97.3–104%, with an RSD of 1.23–7.10%. This method was further employed to detect glucose in diluted serum samples. It was found that the results obtained from our method were close to those provided by the hospital, indicating the practical applicability of this multi-catalytic sensing system (Table 5).

**TABLE 5:** Determination of the glucose concentrations in serum samples.

Samples	The proposed method^a^ in this work (mM)	Glucose meter method^b^ (mM)
Serum 1	5.11 ± 0.016	4.89
Serum 2	5.30 ± 0.041	5.63
Serum 3	6.39 ± 0.032	6.85

a The results of three instances of parallel determination.

b The glucose determination was performed directly in the laboratory for clinical analysis at the School of Huaqiao University

Moreover, DDVP was chosen to further conduct the recovery experiments. Results demonstrated that the recovery of DDVP in various fruit peels, namely, apple skin, pear skin, and peach skin, was found to locate in the range of 99.3–106.4% with acceptable relative standard deviations (RSDs) (Supplementary Table S3). Taken together, these results were supportive of the good reliability of this multi-catalytic sensing system.

## Conclusion

In this work, peroxidase-like CDs were prepared by using various natural amino acids. The kinetic analysis revealed that CD-phenylalanine could possess excellent catalytic activity toward TMB as the substrate in the presence of H_2_O_2_. The catalytic activity of CDs was dependent on pH, temperature, and H_2_O_2_ concentration. The catalysis reaction mechanism of CDs was probed by ESR experiments which indicated that the generated active species HO in the TMB–H_2_O_2_–CD-phenylalanine system should be responsible for the observed catalytic activity. A simple, highly selective, and sensitive colorimetric sensing system for H_2_O_2_ and glucose was thus established, with LODs of 6.50 and 0.84 μM for H_2_O_2_ and glucose, respectively. Furthermore, on the basis of the peroxidase-like activity of CD-phenylalanine, by introducing continuous double enzymatic systems of AChE and CHO, organophosphorus or organochlorine pesticides, such as dimethoate, DDVP, and parathion-methyl, were found to suppress the chromogenic reaction of TMB catalyzed by CD-phenylalanine. Therefore, a simple, facile, and sensitive multi-catalytic sensing system was successfully established for parathion-methyl, DDVP, and dimethoate, with LODs of 7.12, 8.47, and 14.6 nM, respectively. The study of practical application indicated that this sensing strategy has great potential to be developed as applicable biosensors in the future.

## Data Availability

The original contributions presented in the study are included in the article/Supplementary Material; further inquiries can be directed to the corresponding author.
