# Supplementary material for: A Multi-Catalytic Sensing for Hydrogen Peroxide, Glucose, and Organophosphorus Pesticides Based on Carbon Dots
Source: Front Chem. 2021 Jul 12;9:713104. doi: 10.3389/fchem.2021.713104 (PMC8362664; doi:10.3389/fchem.2021.713104)
Supplement: Supplementary file 1 [file datasheet1.pdf]

Supplementary Material

1 Synthesis of CDs

The process of various CDs synthesized was follow: (1) 0.0125 mol amino acids were first dissolved in 36 mL ultrapure water. The mixtures were stirred at room temperature for several minutes and sealed in a 50 mL Teflon-equipped stainless steel autoclave for the following hydrothermal treatment at 200 ^{o}C for 12 h. (2) The obtained suspension was then centrifuged at a 10,000 rpm for 10 min and the supernatants were filtered with a ultrafiltration membrane (MWCO=1000) for 1 h to remove the excess precursors. After filtering through 0.2 μm Teflon filter, a clear aqueous suspension was finally obtained.

2 QY measurements

Reference on QY measurements: Lakowicz, J.R. Principles of Fluorescence Spectroscopy, 2nd Ed., 1999, Kluwer Academic/Plenum Publishers, New York. The QY of the CDs was measured with quinine sulfate as a reference (QY = 0.54 in 0.1 M H2SO4, excited at 360 nm). QY was calculated according to the following equation:

$$QY = QY_R \frac{I}{I_R} \frac{OD_R}{OD} \frac{n^2}{n_R^2}$$

Where QY is the quantum yield, I is the measured integrated emission intensity, n is the refractive index, and OD is the optical density. The subscript R refers to the reference fluorophore of known QY (quinine sulfate used in the work). In order to minimize re-absorption effects, absorbance in the 1 cm fluorescence cuvette were kept under 0.05 at the excitation wavelength.

3 The photographs of CDs

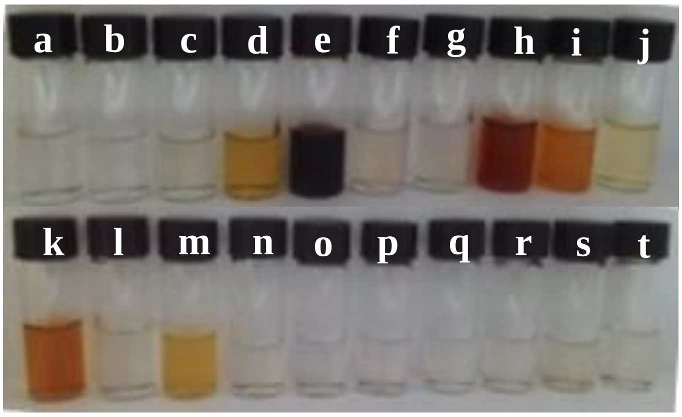

Fig. S1 The photographs of CDs solutions with 20 kinds of raw amino acid synthesized under visible light (The CDs solution. a-t: CDs-aspartic acid, CDs-glutamic, CDs-lysine, CDs-arginine, CDs-histidine, CDs-tryptophan, CDs-tyrosine, CDs-serine, CDs-

cystine, CDs-methionine, CDs-asparaginate, CDs-glntamine, CDs-threonine, CDs-glycine, CDs-alanine, CDs-valine, CDs-leucine, CDs-isoleucine, CDs-phenylalanine and CDs-proline, respectively).

4 UV-vis spectra of CDs

(a)

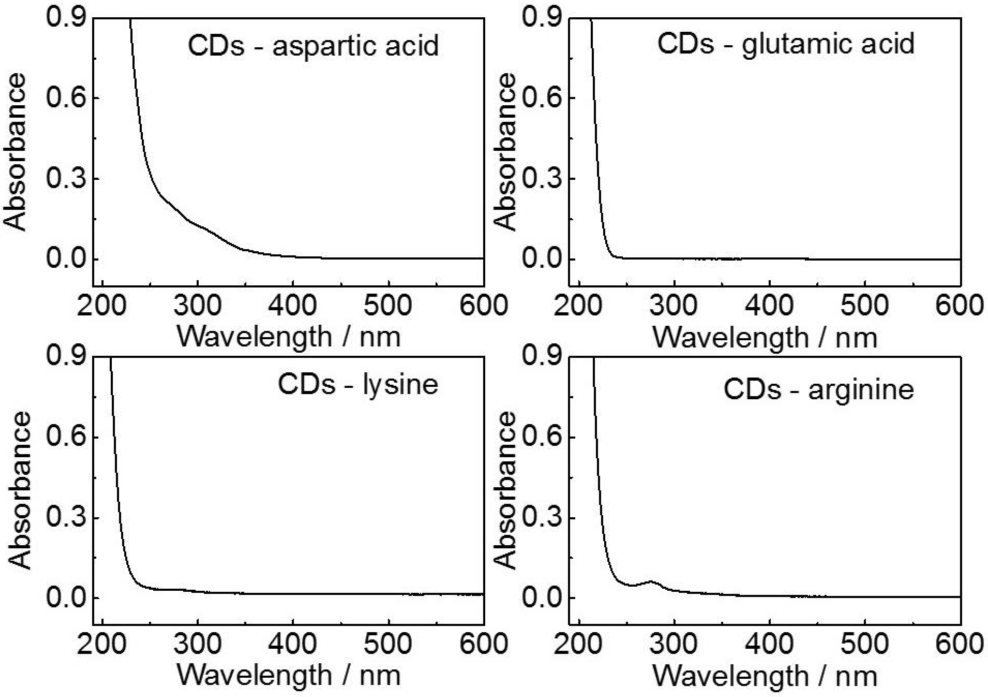

(b)

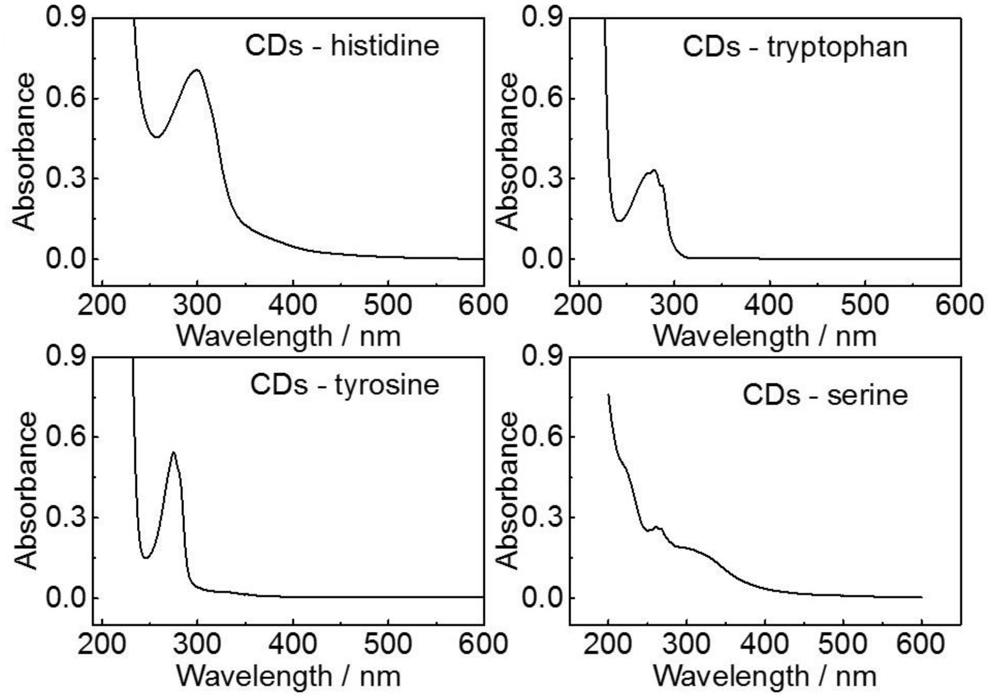

(c)

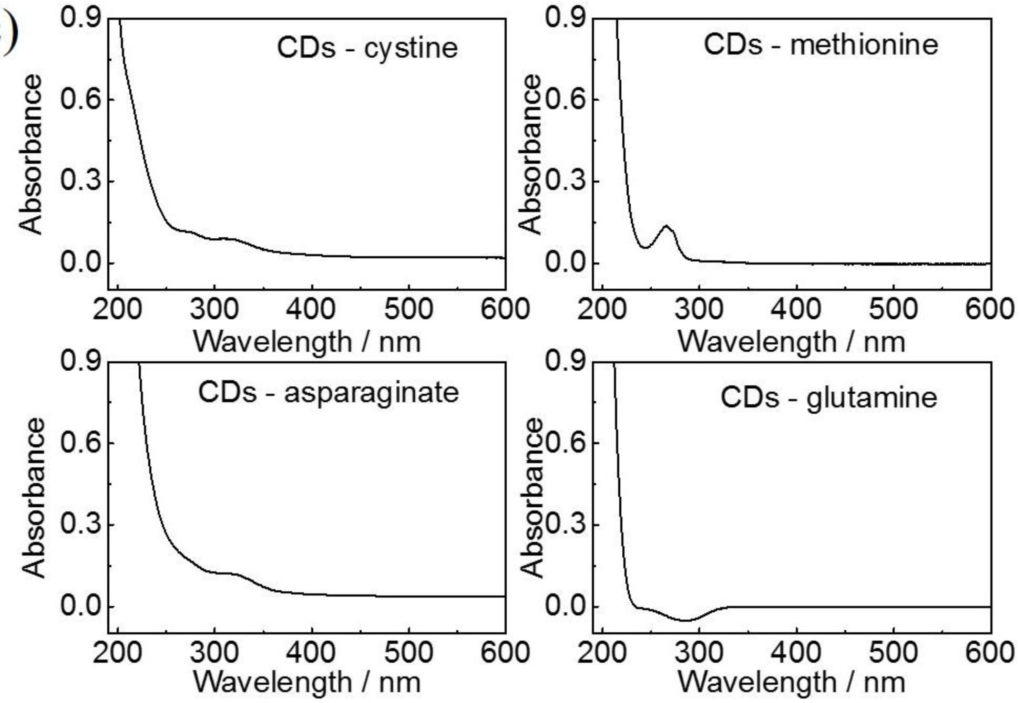
CDs - cystine
CDs - methionine
CDs - asparaginate
CDs - glutamine
Absorbance
Wavelength / nm
0.9
0.6
0.3
0.0
200
300
400
500
600

(d)

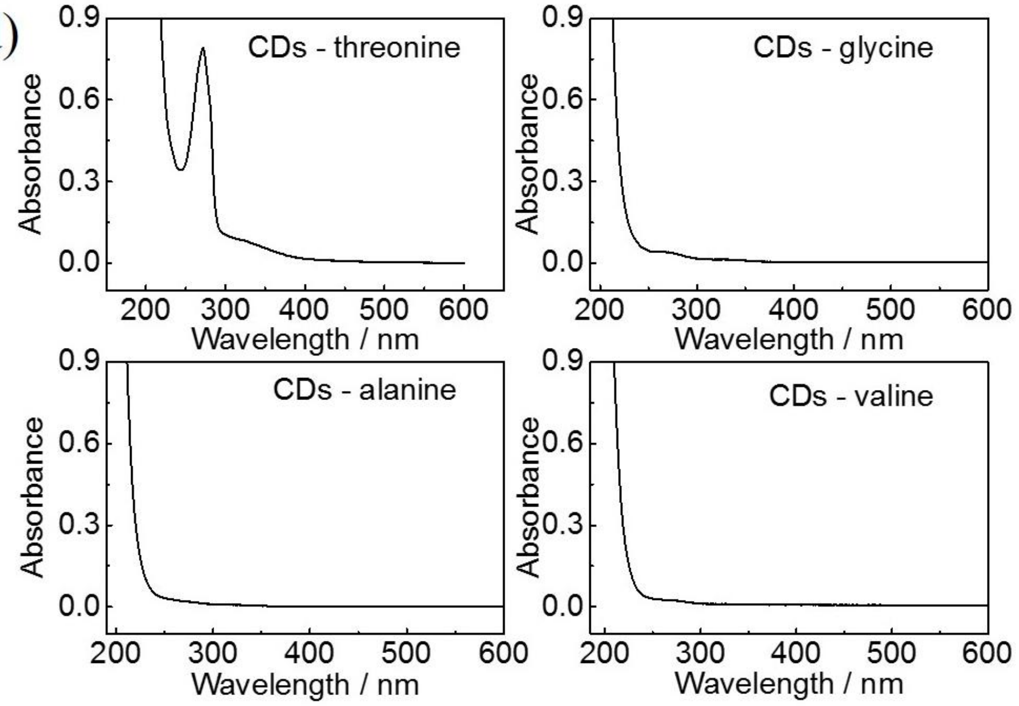
CDs - threonine
CDs - glycine
CDs - alanine
CDs - valine
Absorbance
Wavelength / nm
0.9
0.6
0.3
0.0
200
300
400
500
600

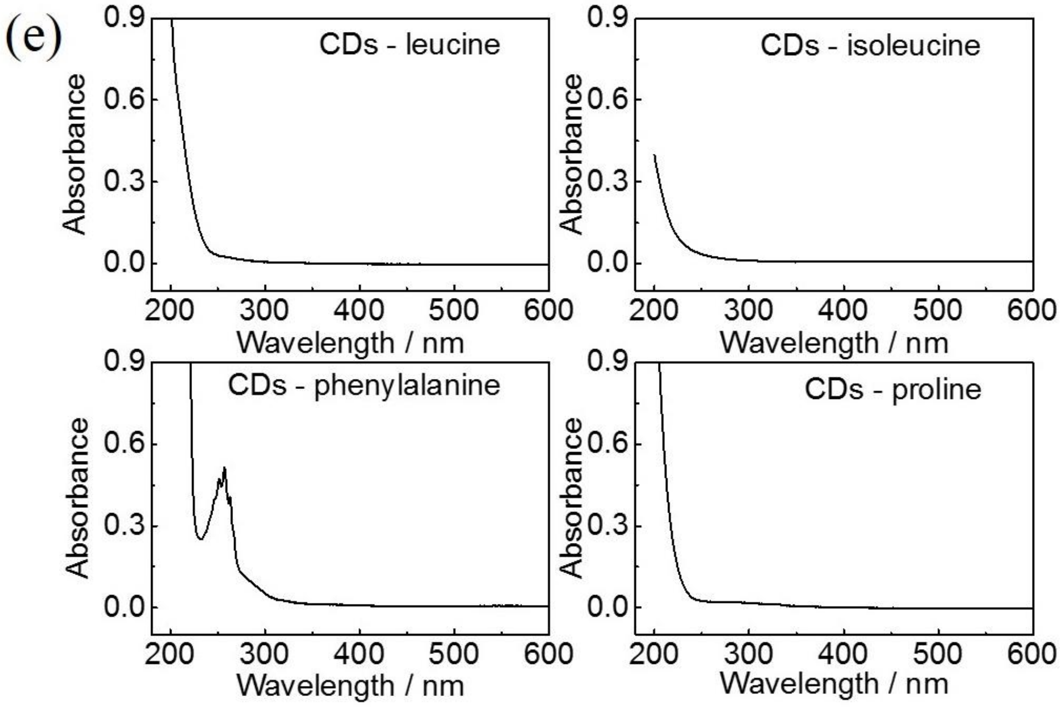

Fig. S2 UV-vis absorption spectra of different CDs

5 PL spectra of CDs

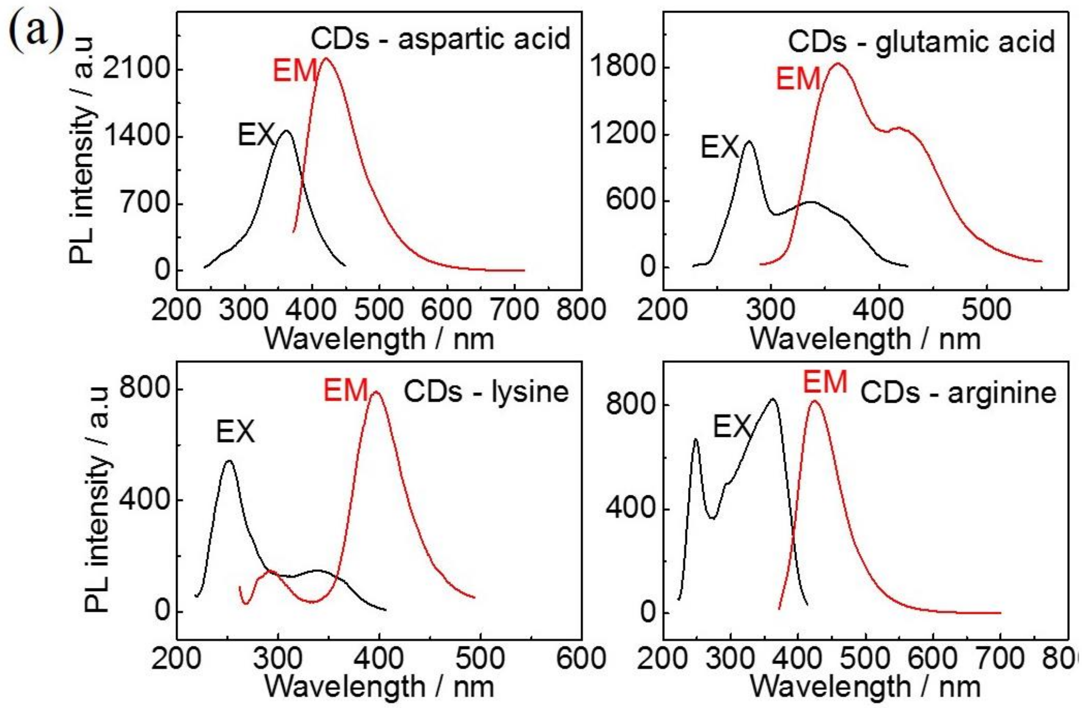

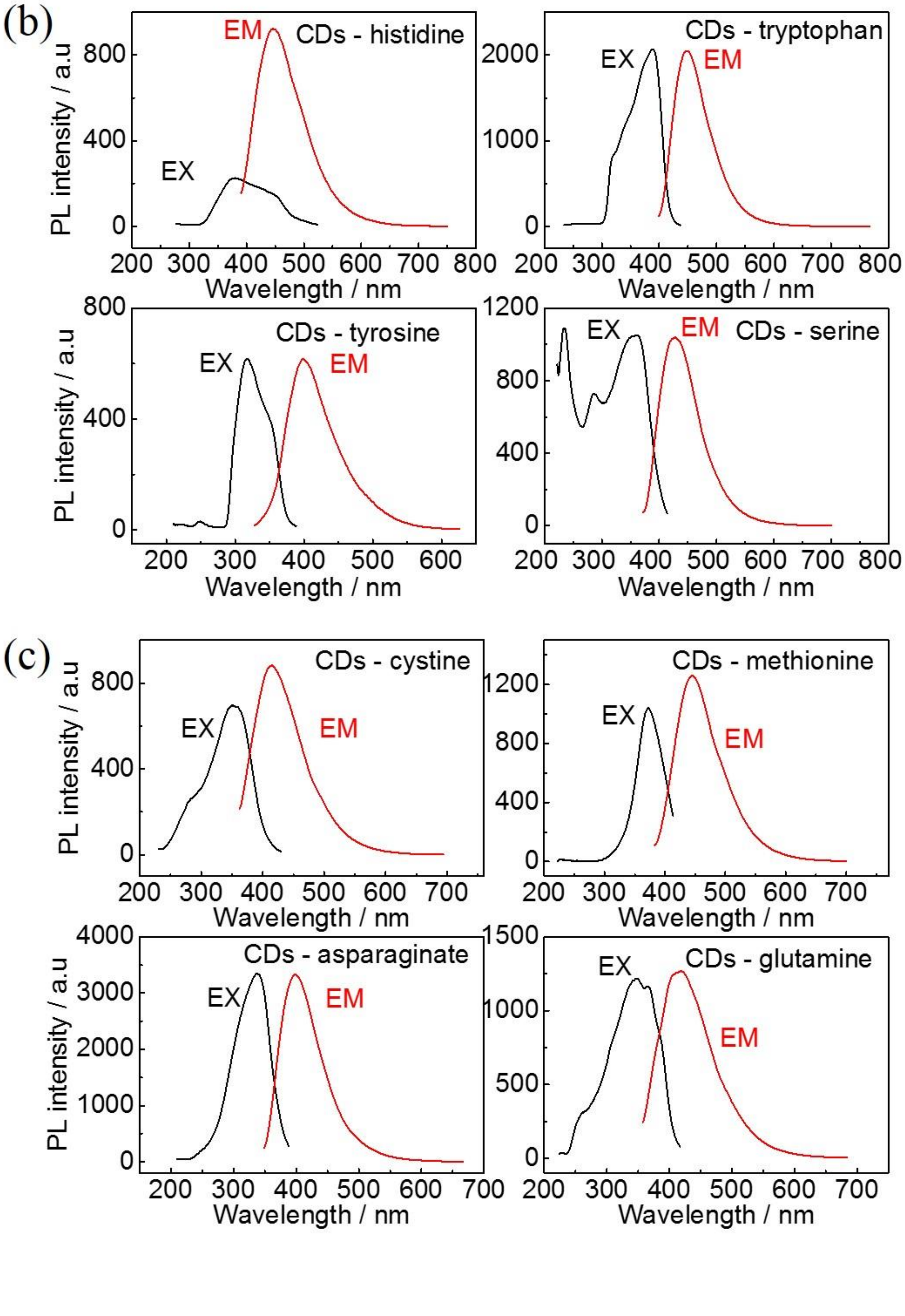
(b)
CDs - histidine
CDs - tryptophan
CDs - tyrosine
CDs - serine
(c)
CDs - cystine
CDs - methionine
CDs - asparaginate
CDs - glutamine
EX
EM
PL intensity / a.u
Wavelength / nm

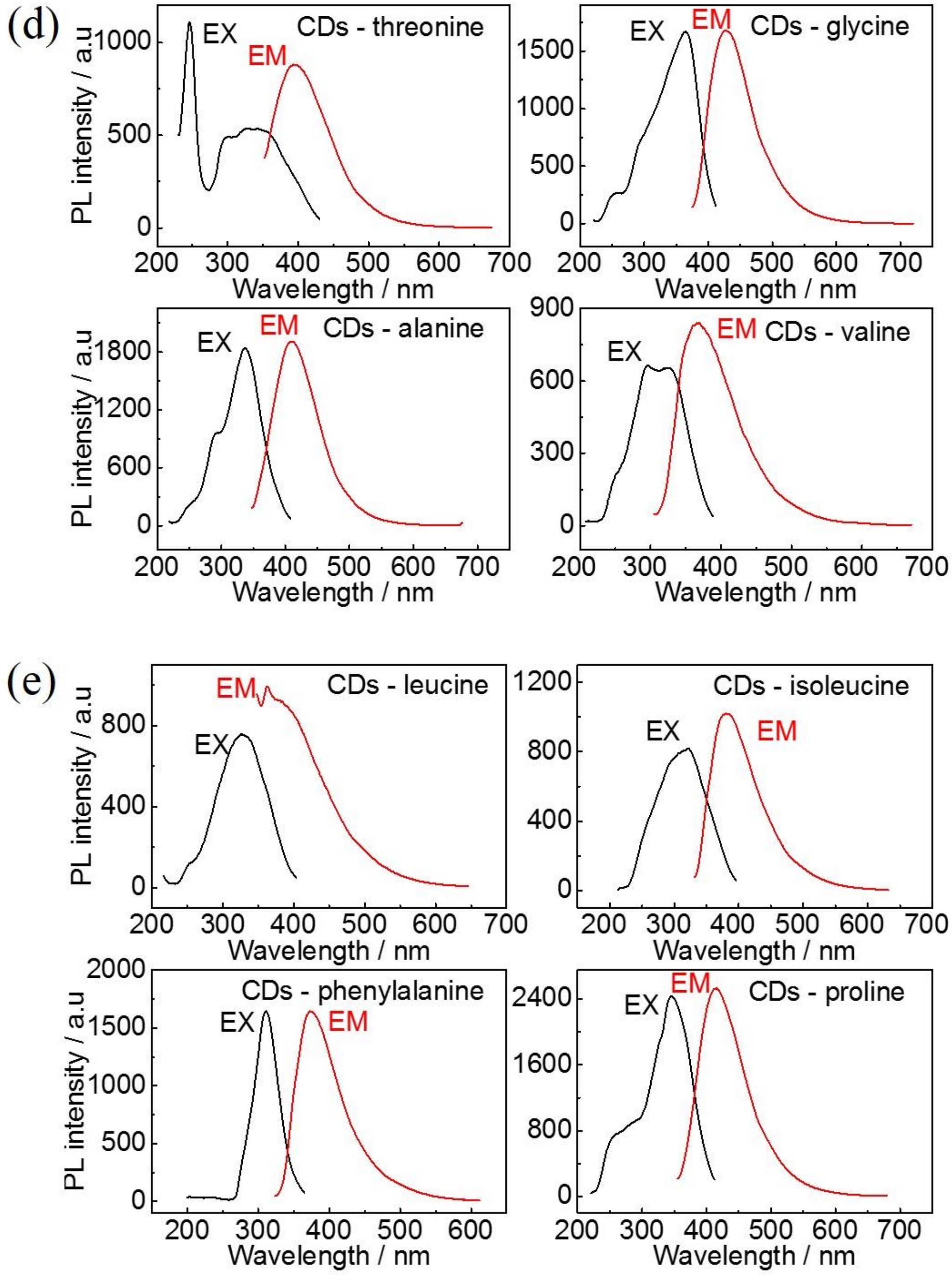

Fig. S3 Photoluminescence spectra of different CDs

6 The optical properties of CDs

Table S1 The optical properties of the synthesized CDs from different amino acids

S. no.	Carbon source	λ_{EX} (nm)	λ_{EM} (nm)	PL QY (%)	Averaged PL lifetime (ns)
1	aspartic acid	362	421	14	5.03 ±0.14
2	glutamic acid	280	419	5.7	8.32 ±0.20
3	lysine	252	395	8.0	6.09 ±0.24
4	arginine	362	425	15	6.00 ±0.25
5	histidine	380	445	5.0	5.63 ±0.04
6	tryptophan	389	449	4.0	8.51 ±0.13
7	tyrosine	318	399	11	2.29 ±0.01
8	serine	362	428	11	9.78 ±0.17
9	cystine	352	415	11	5.98 ±0.08
10	methionine	373	445	6.6	6.26 ±0.15
11	asparagine	338	398	13	8.17 ±0.21
12	glutamine	347	419	9.0	7.07 ±0.06
13	threonine	245	398	15	14.23 ±0.49
14	glycine	365	427	9.3	9.16 ±0.39
15	alanine	338	412	6.9	10.02 ±0.34
16	valine	285	360	5.7	9.48 ±0.22
17	leucine	328	363	3.3	5.05 ±0.12
18	isoleucine	321	380	5	3.08 ±0.07
19	phenylalanine	311	373	9.6	9.63 ±0.15
20	proline	345	416	11	7.14 ±0.18

7 Photoluminescence lifetime of different CDs

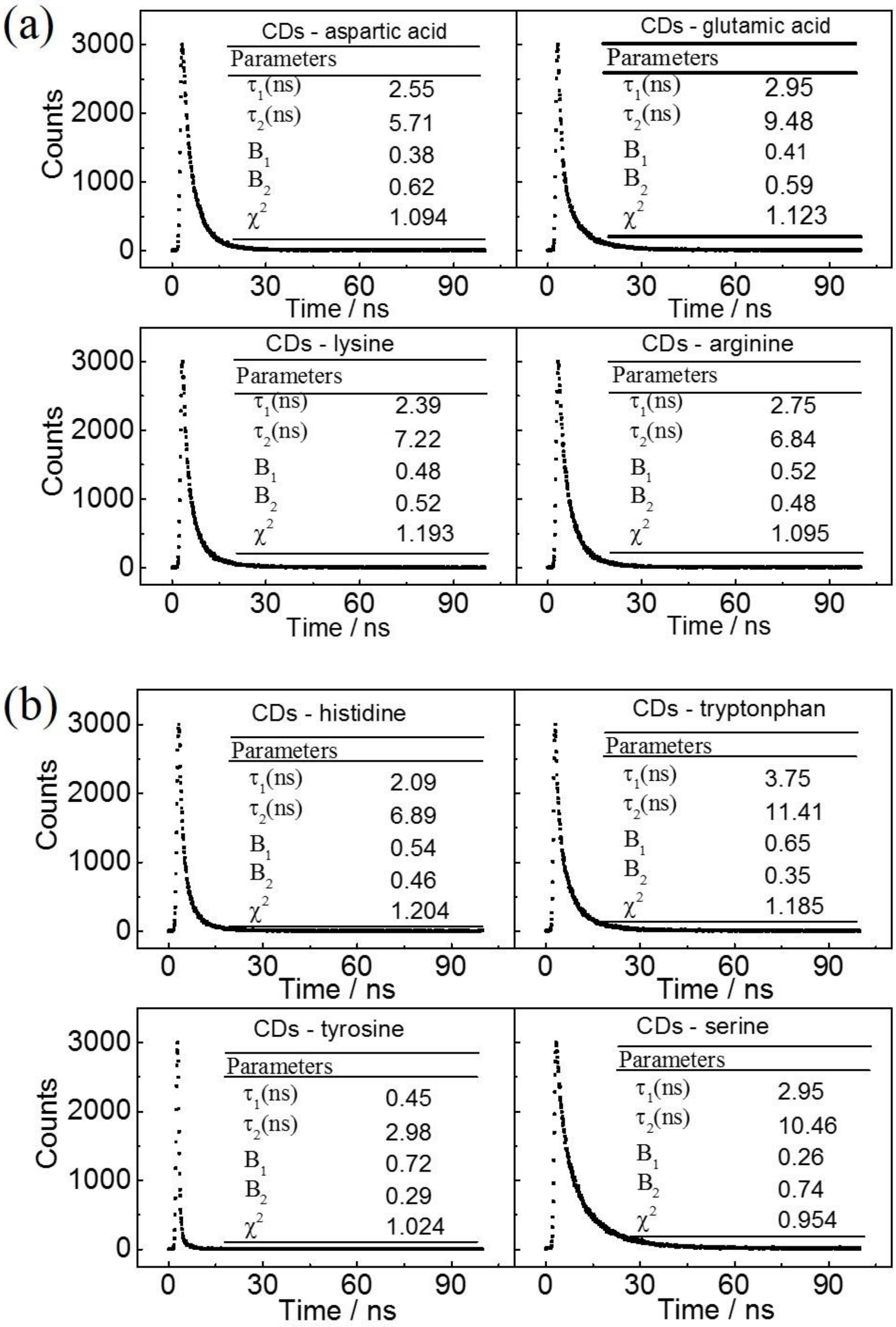

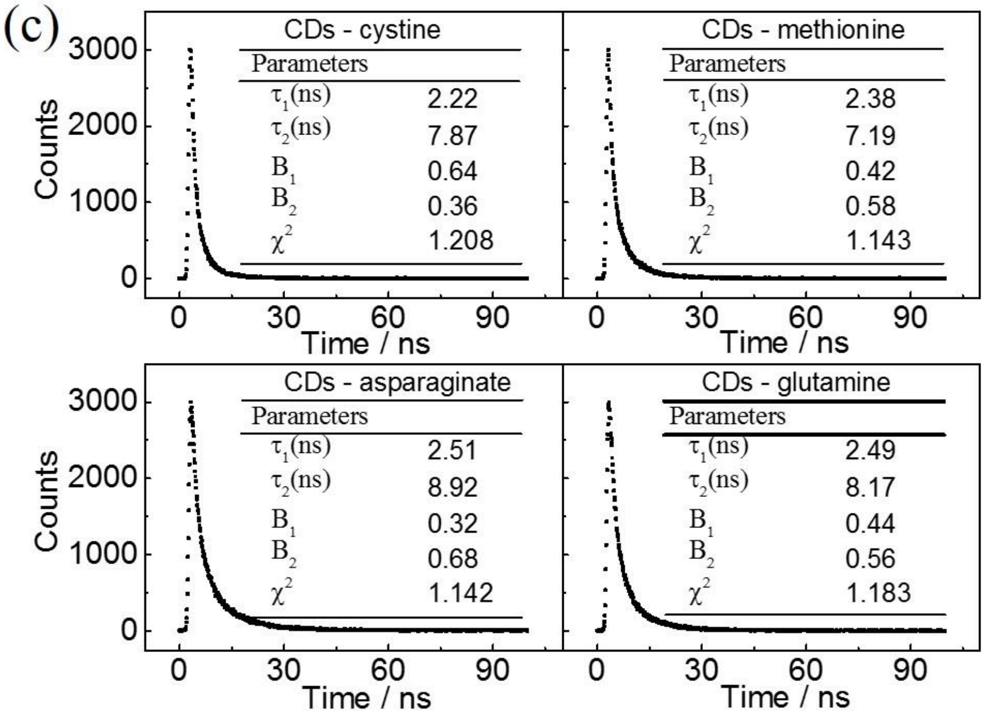
(c)
CDs - cystine
Parameters
τ1(ns) 2.22
τ2(ns) 7.87
B1 0.64
B2 0.36
χ2 1.208
CDs - methionine
Parameters
τ1(ns) 2.38
τ2(ns) 7.19
B1 0.42
B2 0.58
χ2 1.143
CDs - asparaginate
Parameters
τ1(ns) 2.51
τ2(ns) 8.92
B1 0.32
B2 0.68
χ2 1.142
CDs - glutamine
Parameters
τ1(ns) 2.49
τ2(ns) 8.17
B1 0.44
B2 0.56
χ2 1.183
Counts
Time / ns

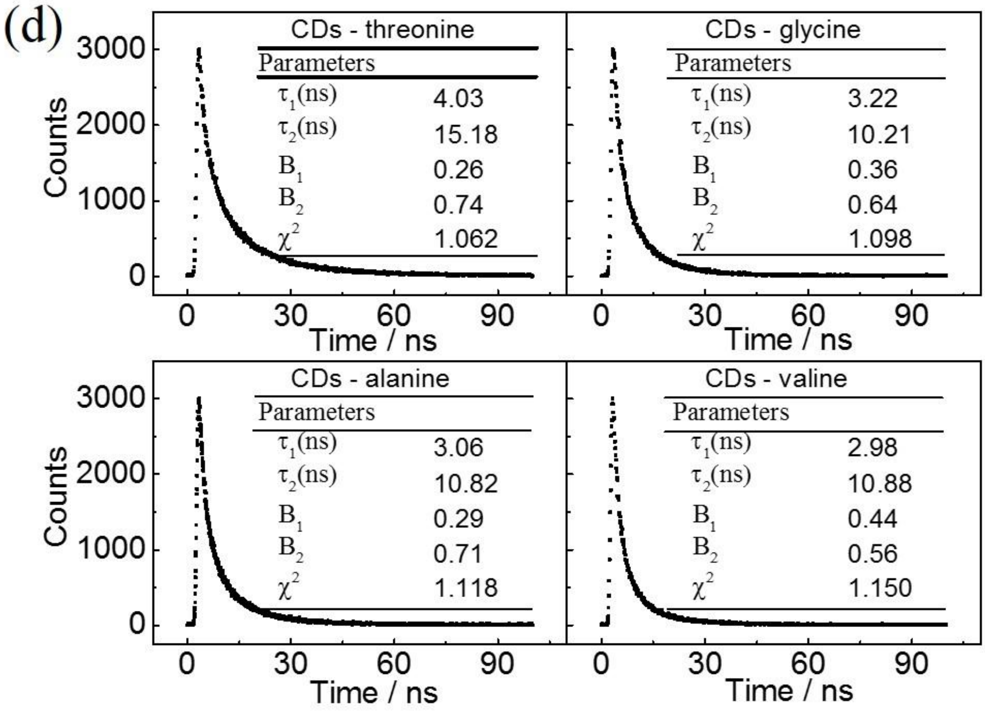
(d)
CDs - threonine
Parameters
τ1(ns) 4.03
τ2(ns) 15.18
B1 0.26
B2 0.74
χ2 1.062
CDs - glycine
Parameters
τ1(ns) 3.22
τ2(ns) 10.21
B1 0.36
B2 0.64
χ2 1.098
CDs - alanine
Parameters
τ1(ns) 3.06
τ2(ns) 10.82
B1 0.29
B2 0.71
χ2 1.118
CDs - valine
Parameters
τ1(ns) 2.98
τ2(ns) 10.88
B1 0.44
B2 0.56
χ2 1.150
Counts
Time / ns

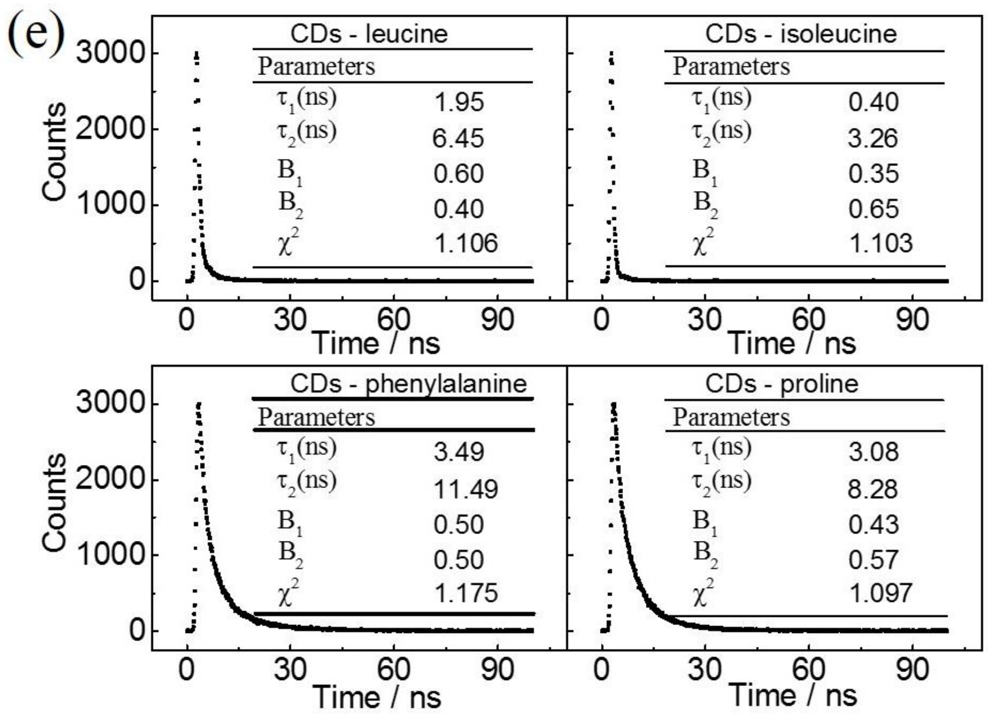

Fig. S4 Photoluminescence lifetime of different CDs

8 Selectivity of glucose detection

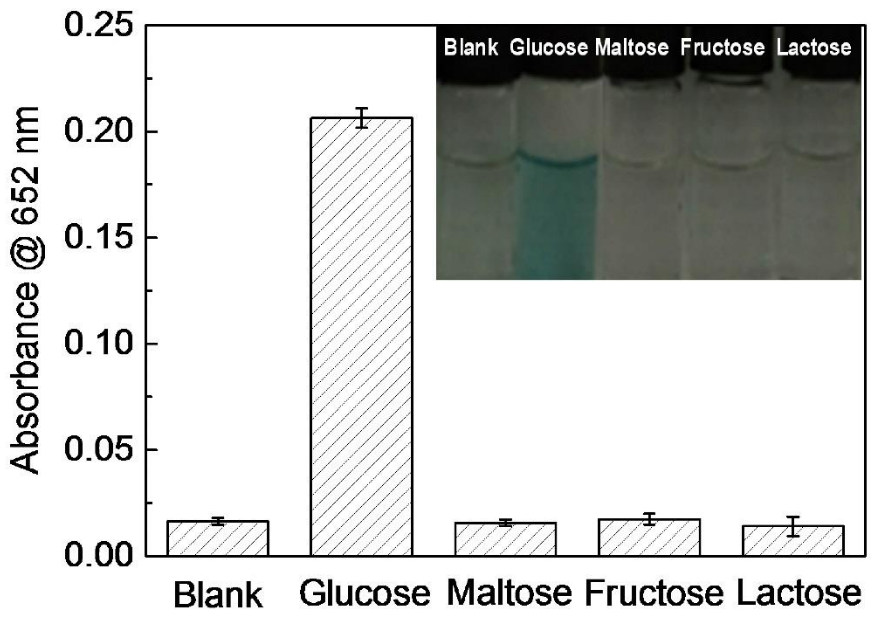

Fig. S5 Selectivity of glucose detection based on the peroxidase-like catalytic activity of the CDs. Absorbance at 652 nm was measured after the addition of CDs for 15 min. Inset: typical photography of glucose detection with the colorimetric method using GOx and CDs as the catalysts. Experiment conditions: [TMB]=0.67 mM, [GOx]=8 μg

mL^{-1}, [glucose]=1 mM, [maltose]=[fructose]=[lactose]=5 mM, 35 °C, 0.2 M NaOAc-HOAc buffer solution (pH 4.2) containing 40 μg mL^{-1} the CDs solution. The error bars represent the standard deviation of three parallel measurements.

9 Standard recovery experiments of the glucose concentrations in fruit samples

Table S2 Standard recovery experiments of the glucose concentrations in fruit samples

Samples	Added value (mM)	Measured value (mM)	Recovery (%)	RSD[a]
Apple juice	16.0	16.5	103.1	1.23 %
	20.0	19.8	99.00	5.30 %
	25.0	24.5	98.00	6.00 %
Orange juice	16.0	16.2	100.1	2.23 %
	20.0	20.4	102.0	4.20 %
	25.0	25.4	101.6	7.10 %
Watermelon juice	16.0	14.6	97.30	5.00 %
	20.0	20.8	104.0	2.20 %
	25.0	25.9	103.6	3.30 %

[a]Note: The results of three times parallel determination.

10 Recovery experiments of DDVP in fruit samples

Table S3 Recovery experiments of DDVP in fruit samples

Samples	Added value (μM)	Measured value (μM)	Recovery (%)	RSD[a]
Apple skin	5.00	5.32	106.4	4.8 %
	15.0	15.1	100.7	7.9 %
Pear skin	5.00	5.28	105.6	5.1 %
	15.0	14.9	99.3	8.2 %
Peach skin	5.00	4.97	99.4	3.5 %
	15.0	15.2	101.3	5.3 %
